# Supplementary material for: Thermal effect on the fecundity and longevity of Bactrocera dorsalis adults and their improved oviposition model
Source: PLoS One. 2020 Jul 15;15(7):e0235910. doi: 10.1371/journal.pone.0235910 (PMC7363081; doi:10.1371/journal.pone.0235910)
Supplement: S1 Table — (DOCX) [file pone.0235910.s001.docx]

**S1 Table. The estimated development rate of *Bactrocera dorsalis* female at various constant temperatures**

| Temperature | Estimated value | |
| --- | --- | --- |
|  | Nonlinear function | Linear function |
| 10 | 0.009835581 | -0.01189231 |
| 10.5 | 0.009857957 | -0.010906276 |
| 11 | 0.00988274 | -0.009920241 |
| 11.5 | 0.009910187 | -0.008934207 |
| 12 | 0.009940586 | -0.007948172 |
| 12.5 | 0.009974254 | -0.006962138 |
| 13 | 0.010011543 | -0.005976103 |
| 13.5 | 0.010052842 | -0.004990069 |
| 14 | 0.010098582 | -0.004004034 |
| 14.5 | 0.010149241 | -0.003018 |
| 15 | 0.010205347 | -0.002031965 |
| 15.5 | 0.010267488 | -0.001045931 |
| 16 | 0.010336311 | -5.9896E-05 |
| 16.5 | 0.010412536 | 0.000926138 |
| 17 | 0.010496957 | 0.001912173 |
| 17.5 | 0.010590458 | 0.002898208 |
| 18 | 0.010694013 | 0.003884242 |
| 18.5 | 0.010808705 | 0.004870276 |
| 19 | 0.01093573 | 0.005856311 |
| 19.5 | 0.011076416 | 0.006842346 |
| 20 | 0.011232232 | 0.00782838 |
| 20.5 | 0.011404803 | 0.008814414 |
| 21 | 0.011595934 | 0.009800449 |
| 21.5 | 0.011807618 | 0.010786484 |
| 22 | 0.012042067 | 0.011772518 |
| 22.5 | 0.012301728 | 0.012758553 |
| 23 | 0.012589314 | 0.013744587 |
| 23.5 | 0.012907826 | 0.014730622 |
| 24 | 0.013260592 | 0.015716656 |
| 24.5 | 0.013651293 | 0.016702691 |
| 25 | 0.014084011 | 0.017688725 |
| 25.5 | 0.014563264 | 0.01867476 |
| 26 | 0.015094055 | 0.019660794 |
| 26.5 | 0.015681927 | 0.020646829 |
| 27 | 0.01633302 | 0.021632863 |
| 27.5 | 0.017054131 | 0.022618898 |
| 28 | 0.01785279 | 0.023604932 |
| 28.5 | 0.018737337 | 0.024590967 |
| 29 | 0.019717008 | 0.025577001 |
| 29.5 | 0.020802034 | 0.026563036 |
| 30 | 0.022003743 | 0.02754907 |
| 30.5 | 0.023334684 | 0.028535105 |
| 31 | 0.024808754 | 0.029521139 |
| 31.5 | 0.026441346 | 0.030507174 |
| 32 | 0.028249507 | 0.031493208 |
| 32.5 | 0.030252118 | 0.032479243 |
| 33 | 0.03247009 | 0.033465277 |
| 33.5 | 0.034926582 | 0.034451312 |
| 34 | 0.037647246 | 0.035437346 |
| 34.5 | 0.040660491 | 0.036423381 |
| 35 | 0.04399778 | 0.037409415 |
| 35.1 | 0.044707126 | 0.037606622 |
| 36 | 0.05178763 | 0.039381484 |
| 36.5 | 0.056321532 | 0.040367519 |
| 37 | 0.061343011 | 0.041353553 |
| 37.5 | 0.0669045 | 0.042339588 |
| 38 | 0.073064072 | 0.043325622 |
| 38.5 | 0.079886044 | 0.044311657 |
| 39 | 0.087441652 | 0.045297691 |
| 39.5 | 0.095809791 | 0.046283726 |
| 40 | 0.10507784 | 0.04726976 |
