# Supplementary material for: Thermal effect on the fecundity and longevity of Bactrocera dorsalis adults and their improved oviposition model
Source: PLoS One. 2020 Jul 15;15(7):e0235910. doi: 10.1371/journal.pone.0235910 (PMC7363081; doi:10.1371/journal.pone.0235910)
Supplement: S2 Table — (DOCX) [file pone.0235910.s002.docx]

**S2 Table. The estimated development rate of *Bactrocera dorsalis* male at various constant temperatures**

| Temperature | Estimated value | |
| --- | --- | --- |
|  | Nonlinear function | Linear function |
| 10 | 0.009776944 | -0.01322577 |
| 10.5 | 0.009819394 | -0.012072112 |
| 11 | 0.009865922 | -0.010918453 |
| 11.5 | 0.009916917 | -0.009764795 |
| 12 | 0.009972809 | -0.008611136 |
| 12.5 | 0.010034068 | -0.007457478 |
| 13 | 0.01010121 | -0.006303819 |
| 13.5 | 0.010174798 | -0.005150161 |
| 14 | 0.010255454 | -0.003996502 |
| 14.5 | 0.010343855 | -0.002842844 |
| 15 | 0.010440744 | -0.001689185 |
| 15.5 | 0.010546937 | -0.000535527 |
| 16 | 0.010663328 | 0.000618132 |
| 16.5 | 0.010790895 | 0.001771791 |
| 17 | 0.010930712 | 0.002925449 |
| 17.5 | 0.011083955 | 0.004079108 |
| 18 | 0.011251914 | 0.005232766 |
| 18.5 | 0.011436001 | 0.006386425 |
| 19 | 0.011637765 | 0.007540083 |
| 19.5 | 0.011858904 | 0.008693742 |
| 20 | 0.012101279 | 0.0098474 |
| 20.5 | 0.012366927 | 0.011001059 |
| 21 | 0.012658085 | 0.012154717 |
| 21.5 | 0.012977202 | 0.013308376 |
| 22 | 0.013326962 | 0.014462034 |
| 22.5 | 0.013710309 | 0.015615693 |
| 23 | 0.014130467 | 0.016769351 |
| 23.5 | 0.014590972 | 0.01792301 |
| 24 | 0.015095697 | 0.019076668 |
| 24.5 | 0.015648889 | 0.020230327 |
| 25 | 0.016255202 | 0.021383985 |
| 25.5 | 0.016919737 | 0.022537644 |
| 26 | 0.017648085 | 0.023691302 |
| 26.5 | 0.018446374 | 0.024844961 |
| 27 | 0.01932132 | 0.025998619 |
| 27.5 | 0.020280284 | 0.027152278 |
| 28 | 0.021331334 | 0.028305936 |
| 28.5 | 0.022483312 | 0.029459595 |
| 29 | 0.023745911 | 0.030613253 |
| 29.5 | 0.025129753 | 0.031766912 |
| 30 | 0.02664648 | 0.03292057 |
| 30.5 | 0.028308853 | 0.034074229 |
| 31 | 0.030130858 | 0.035227887 |
| 31.5 | 0.032127824 | 0.036381546 |
| 32 | 0.034316551 | 0.037535204 |
| 32.5 | 0.036715454 | 0.038688863 |
| 33 | 0.039344716 | 0.039842521 |
| 33.5 | 0.042226456 | 0.04099618 |
| 34 | 0.045384919 | 0.042149838 |
| 34.5 | 0.048846679 | 0.043303497 |
| 35 | 0.052640859 | 0.044457155 |
| 35.1 | 0.053442343 | 0.044687887 |
| 36 | 0.061357232 | 0.046764472 |
| 36.5 | 0.066352757 | 0.047918131 |
| 37 | 0.071827984 | 0.049071789 |
| 37.5 | 0.077828979 | 0.050225448 |
| 38 | 0.084406227 | 0.051379106 |
| 38.5 | 0.091615066 | 0.052532765 |
| 39 | 0.099516145 | 0.053686423 |
| 39.5 | 0.108175936 | 0.054840082 |
| 40 | 0.117667296 | 0.05599374 |
