# Supplementary material for: Thermal effect on the fecundity and longevity of Bactrocera dorsalis adults and their improved oviposition model
Source: PLoS One. 2020 Jul 15;15(7):e0235910. doi: 10.1371/journal.pone.0235910 (PMC7363081; doi:10.1371/journal.pone.0235910)
Supplement: S3 Table — (DOCX) [file pone.0235910.s003.docx]

**S3 Table. The physiological age and survival probability of *Bactrocera dorsalis* female at various constant temperatures**

| Temperature | Physiological age | Survival probability |
| --- | --- | --- |
| 13 | 0 | 1 |
|  | 0.255159475 | 1 |
|  | 0.265791119 | 0.941176471 |
|  | 0.318949343 | 0.882352941 |
|  | 0.499687305 | 0.823529412 |
|  | 0.669793621 | 0.705882353 |
|  | 0.754846779 | 0.647058824 |
|  | 0.797373358 | 0.588235294 |
|  | 0.839899937 | 0.529411765 |
|  | 0.924953096 | 0.470588235 |
|  | 1.052532833 | 0.411764706 |
|  | 1.307692308 | 0.176470588 |
|  | 1.562851782 | 0.117647059 |
|  | 1.775484678 | 0 |
| 16 | 0.130841121 | 1 |
|  | 0.14953271 | 0.95 |
|  | 0.61682243 | 0.9 |
|  | 0.635514019 | 0.85 |
|  | 0.672897196 | 0.8 |
|  | 0.691588785 | 0.75 |
|  | 0.747663551 | 0.7 |
|  | 0.785046729 | 0.65 |
|  | 0.859813084 | 0.6 |
|  | 0.971962617 | 0.55 |
|  | 1.046728972 | 0.45 |
|  | 1.177570093 | 0.4 |
|  | 1.196261682 | 0.3 |
|  | 1.289719626 | 0.25 |
|  | 1.308411215 | 0.2 |
|  | 1.401869159 | 0.1 |
|  | 1.588785047 | 0 |
| 20 | 0.607703281 | 1 |
|  | 0.616262482 | 0.944444444 |
|  | 0.650499287 | 0.888888889 |
|  | 0.667617689 | 0.833333333 |
|  | 0.718972896 | 0.777777778 |
|  | 0.736091298 | 0.722222222 |
|  | 0.873038516 | 0.666666667 |
|  | 0.890156919 | 0.611111111 |
|  | 0.907275321 | 0.555555556 |
|  | 0.924393723 | 0.5 |
|  | 1.018544936 | 0.388888889 |
|  | 1.215406562 | 0.333333333 |
|  | 1.249643367 | 0.277777778 |
|  | 1.27532097 | 0.222222222 |
|  | 1.300998573 | 0.166666667 |
|  | 1.335235378 | 0.111111111 |
|  | 1.35235378 | 0.055555556 |
|  | 1.403708987 | 0 |
| 24 | 0.318674315 | 1 |
|  | 0.331421287 | 0.95 |
|  | 0.599107712 | 0.9 |
|  | 0.611854685 | 0.85 |
|  | 0.713830465 | 0.8 |
|  | 0.752071383 | 0.75 |
|  | 0.790312301 | 0.7 |
|  | 0.803059273 | 0.65 |
|  | 0.841300191 | 0.6 |
|  | 0.917782027 | 0.5 |
|  | 0.956022945 | 0.45 |
|  | 0.994263862 | 0.4 |
|  | 1.172721479 | 0.3 |
|  | 1.236456342 | 0.25 |
|  | 1.249203314 | 0.2 |
|  | 1.351179095 | 0.15 |
|  | 1.427660931 | 0.1 |
|  | 1.606118547 | 0.05 |
|  | 1.810070108 | 0 |
| 28 | 0.439306358 | 1 |
|  | 0.462427746 | 0.875 |
|  | 0.647398844 | 0.8125 |
|  | 0.670520231 | 0.75 |
|  | 0.693641618 | 0.6875 |
|  | 0.855491329 | 0.625 |
|  | 0.878612717 | 0.5625 |
|  | 0.924855491 | 0.5 |
|  | 0.994219653 | 0.375 |
|  | 1.225433526 | 0.3125 |
|  | 1.248554913 | 0.3125 |
|  | 1.271676301 | 0.25 |
|  | 1.410404624 | 0.1875 |
|  | 1.595375723 | 0.125 |
|  | 1.641618497 | 0 |
| 32 | 0.166201117 | 1 |
|  | 0.189944134 | 0.941176471 |
|  | 0.427374302 | 0.882352941 |
|  | 0.664804469 | 0.823529412 |
|  | 0.712290503 | 0.764705882 |
|  | 0.80726257 | 0.705882353 |
|  | 0.87849162 | 0.588235294 |
|  | 0.925977654 | 0.470588235 |
|  | 0.973463687 | 0.411764706 |
|  | 1.092178771 | 0.352941176 |
|  | 1.187150838 | 0.235294118 |
|  | 1.424581006 | 0.176470588 |
|  | 1.472067039 | 0.117647059 |
|  | 1.519553073 | 0.058823529 |
|  | 2.136871508 | 0 |
| 35 | 0.535433071 | 1 |
|  | 0.580052493 | 0.941176471 |
|  | 0.624671916 | 0.823529412 |
|  | 0.669291339 | 0.764705882 |
|  | 0.758530184 | 0.705882353 |
|  | 0.892388451 | 0.647058824 |
|  | 0.937007874 | 0.588235294 |
|  | 1.026246719 | 0.411764706 |
|  | 1.204724409 | 0.235294118 |
|  | 1.3832021 | 0.176470588 |
|  | 1.427821522 | 0.117647059 |
|  | 1.472440945 | 0.058823529 |
|  | 1.695538058 | 0 |
