# Supplementary material for: Thermal effect on the fecundity and longevity of Bactrocera dorsalis adults and their improved oviposition model
Source: PLoS One. 2020 Jul 15;15(7):e0235910. doi: 10.1371/journal.pone.0235910 (PMC7363081; doi:10.1371/journal.pone.0235910)
Supplement: S4 Table — (DOCX) [file pone.0235910.s004.docx]

**S4 Table. The estimated survival probability of *Bactrocera dorsalis* female**

| Physiological age | Estimated survival probability |
| --- | --- |
| 0 | 1 |
| 0.1 | 0.99911771 |
| 0.2 | 0.993241189 |
| 0.3 | 0.9778943 |
| 0.4 | 0.949228985 |
| 0.5 | 0.904427991 |
| 0.6 | 0.842192477 |
| 0.7 | 0.763160237 |
| 0.8 | 0.670097523 |
| 0.9 | 0.567733513 |
| 1 | 0.462184113 |
| 1.1 | 0.360031906 |
| 1.2 | 0.267254201 |
| 1.3 | 0.188268333 |
| 1.4 | 0.125348603 |
| 1.5 | 0.078556023 |
| 1.6 | 0.046152252 |
| 1.7 | 0.025316417 |
| 1.8 | 0.012913871 |
| 1.9 | 0.006101132 |
| 2 | 0.002659039 |
| 2.1 | 0.001064793 |
| 2.2 | 0.000390214 |
| 2.3 | 0.000130351 |
| 2.4 | 3.95346E-05 |
| 2.5 | 1.08437E-05 |
