# Supplementary material for: Thermal effect on the fecundity and longevity of Bactrocera dorsalis adults and their improved oviposition model
Source: PLoS One. 2020 Jul 15;15(7):e0235910. doi: 10.1371/journal.pone.0235910 (PMC7363081; doi:10.1371/journal.pone.0235910)
Supplement: S5 Table — (DOCX) [file pone.0235910.s005.docx]

**S5 Table. The physiological age and survival probability of *Bactrocera dorsalis* male at various constant temperatures**

| Temperature | Physiological age | Survival probability |
| --- | --- | --- |
| 13 | 0 | 1 |
|  | 0.433628319 | 1 |
|  | 0.442477876 | 0.958333333 |
|  | 0.477876106 | 0.916666667 |
|  | 0.654867257 | 0.875 |
|  | 0.725663717 | 0.833333333 |
|  | 0.796460177 | 0.791666667 |
|  | 0.902654867 | 0.666666667 |
|  | 0.938053097 | 0.541666667 |
|  | 1.079646018 | 0.416666667 |
|  | 1.115044248 | 0.333333333 |
|  | 1.150442478 | 0.291666667 |
|  | 1.185840708 | 0.208333333 |
|  | 1.221238938 | 0.125 |
|  | 1.292035398 | 0.041666667 |
|  | 1.362831858 | 0 |
| 16 | 0.116666667 | 1 |
|  | 0.136111111 | 0.952380952 |
|  | 0.408333333 | 0.904761905 |
|  | 0.777777778 | 0.857142857 |
|  | 0.836111111 | 0.761904762 |
|  | 0.913888889 | 0.714285714 |
|  | 0.991666667 | 0.666666667 |
|  | 1.030555556 | 0.571428571 |
|  | 1.05 | 0.476190476 |
|  | 1.069444444 | 0.333333333 |
|  | 1.088888889 | 0.285714286 |
|  | 1.205555556 | 0.238095238 |
|  | 1.225 | 0.142857143 |
|  | 1.322222222 | 0.047619048 |
|  | 1.341666667 | 0 |
| 20 | 0.377113134 | 1 |
|  | 0.389683572 | 0.965517241 |
|  | 0.465106199 | 0.931034483 |
|  | 0.502817512 | 0.862068966 |
|  | 0.527958388 | 0.827586207 |
|  | 0.540528825 | 0.793103448 |
|  | 0.565669701 | 0.75862069 |
|  | 0.703944517 | 0.724137931 |
|  | 0.804508019 | 0.689655172 |
|  | 0.829648895 | 0.620689655 |
|  | 0.892501084 | 0.586206897 |
|  | 0.917641959 | 0.551724138 |
|  | 0.96792371 | 0.482758621 |
|  | 0.980494148 | 0.448275862 |
|  | 0.993064586 | 0.413793103 |
|  | 1.005635024 | 0.379310345 |
|  | 1.043346337 | 0.344827586 |
|  | 1.055916775 | 0.310344828 |
|  | 1.106198526 | 0.275862069 |
|  | 1.219332466 | 0.24137931 |
|  | 1.269614218 | 0.206896552 |
|  | 1.282184655 | 0.172413793 |
|  | 1.395318596 | 0.137931034 |
|  | 1.521022974 | 0.103448276 |
|  | 1.785002167 | 0.068965517 |
|  | 1.910706545 | 0.034482759 |
|  | 2.048981361 | 0 |
| 24 | 0.470204082 | 1 |
|  | 0.484897959 | 0.972222222 |
|  | 0.573061224 | 0.944444444 |
|  | 0.587755102 | 0.916666667 |
|  | 0.60244898 | 0.861111111 |
|  | 0.646530612 | 0.805555556 |
|  | 0.675918367 | 0.777777778 |
|  | 0.72 | 0.75 |
|  | 0.734693878 | 0.722222222 |
|  | 0.793469388 | 0.694444444 |
|  | 0.808163265 | 0.666666667 |
|  | 0.822857143 | 0.638888889 |
|  | 0.83755102 | 0.611111111 |
|  | 0.852244898 | 0.583333333 |
|  | 0.911020408 | 0.5 |
|  | 0.955102041 | 0.472222222 |
|  | 0.969795918 | 0.444444444 |
|  | 1.028571429 | 0.416666667 |
|  | 1.043265306 | 0.388888889 |
|  | 1.072653061 | 0.361111111 |
|  | 1.087346939 | 0.333333333 |
|  | 1.102040816 | 0.277777778 |
|  | 1.146122449 | 0.194444444 |
|  | 1.263673469 | 0.166666667 |
|  | 1.337142857 | 0.138888889 |
|  | 1.484081633 | 0.111111111 |
|  | 1.616326531 | 0.083333333 |
|  | 1.748571429 | 0.055555556 |
|  | 1.822040816 | 0 |
| 28 | 0.438461538 | 1 |
|  | 0.461538462 | 0.966666667 |
|  | 0.484615385 | 0.933333333 |
|  | 0.576923077 | 0.9 |
|  | 0.715384615 | 0.866666667 |
|  | 0.738461538 | 0.833333333 |
|  | 0.761538462 | 0.766666667 |
|  | 0.807692308 | 0.733333333 |
|  | 0.830769231 | 0.7 |
|  | 0.923076923 | 0.666666667 |
|  | 0.946153846 | 0.566666667 |
|  | 0.969230769 | 0.533333333 |
|  | 1.015384615 | 0.5 |
|  | 1.038461538 | 0.466666667 |
|  | 1.084615385 | 0.333333333 |
|  | 1.130769231 | 0.3 |
|  | 1.153846154 | 0.266666667 |
|  | 1.2 | 0.233333333 |
|  | 1.223076923 | 0.166666667 |
|  | 1.246153846 | 0.1 |
|  | 1.361538462 | 0.066666667 |
|  | 1.430769231 | 0.033333333 |
|  | 1.523076923 | 0 |
| 32 | 0.387855044 | 1 |
|  | 0.420176298 | 0.96969697 |
|  | 0.517140059 | 0.909090909 |
|  | 0.61410382 | 0.878787879 |
|  | 0.646425073 | 0.848484848 |
|  | 0.678746327 | 0.818181818 |
|  | 0.743388834 | 0.757575758 |
|  | 0.775710088 | 0.666666667 |
|  | 0.808031342 | 0.606060606 |
|  | 0.872673849 | 0.575757576 |
|  | 0.904995103 | 0.545454545 |
|  | 0.937316357 | 0.484848485 |
|  | 0.96963761 | 0.454545455 |
|  | 1.066601371 | 0.333333333 |
|  | 1.163565132 | 0.303030303 |
|  | 1.195886386 | 0.272727273 |
|  | 1.22820764 | 0.242424242 |
|  | 1.325171401 | 0.212121212 |
|  | 1.357492654 | 0.181818182 |
|  | 1.389813908 | 0.151515152 |
|  | 1.454456415 | 0.121212121 |
|  | 1.486777669 | 0.090909091 |
|  | 1.551420176 | 0.060606061 |
|  | 1.58374143 | 0 |
| 35 | 0.630602782 | 1 |
|  | 0.683153014 | 0.941176471 |
|  | 0.735703246 | 0.911764706 |
|  | 0.788253478 | 0.882352941 |
|  | 0.840803709 | 0.735294118 |
|  | 0.893353941 | 0.558823529 |
|  | 0.945904173 | 0.529411765 |
|  | 0.998454405 | 0.5 |
|  | 1.051004637 | 0.352941176 |
|  | 1.103554869 | 0.264705882 |
|  | 1.1561051 | 0.176470588 |
|  | 1.208655332 | 0.088235294 |
|  | 1.261205564 | 0.058823529 |
|  | 1.313755796 | 0.029411765 |
|  | 1.366306028 | 0 |
