# Supplementary material for: Thermal effect on the fecundity and longevity of Bactrocera dorsalis adults and their improved oviposition model
Source: PLoS One. 2020 Jul 15;15(7):e0235910. doi: 10.1371/journal.pone.0235910 (PMC7363081; doi:10.1371/journal.pone.0235910)
Supplement: S6 Table — (DOCX) [file pone.0235910.s006.docx]

**S6 Table. The estimated survival probability of *Bactrocera dorsalis* male**

| Physiological age | Estimated survival probability |
| --- | --- |
| 0 | 1 |
| 0.1 | 0.999868156 |
| 0.2 | 0.998197333 |
| 0.3 | 0.991698735 |
| 0.4 | 0.975611982 |
| 0.5 | 0.944291624 |
| 0.6 | 0.892195168 |
| 0.7 | 0.815374502 |
| 0.8 | 0.71328966 |
| 0.9 | 0.590367575 |
| 1 | 0.456401594 |
| 1.1 | 0.324980968 |
| 1.2 | 0.209933818 |
| 1.3 | 0.121050947 |
| 1.4 | 0.061233951 |
| 1.5 | 0.026678996 |
| 1.6 | 0.009818839 |
| 1.7 | 0.002990729 |
| 1.8 | 0.000737891 |
| 1.9 | 0.000144192 |
| 2 | 2.17989E-05 |
| 2.1 | 2.48802E-06 |
| 2.2 | 2.09007E-07 |
| 2.3 | 1.25866E-08 |
| 2.4 | 5.28729E-10 |
| 2.5 | 1.50615E-11 |
