# Supplementary material for: Thermal effect on the fecundity and longevity of Bactrocera dorsalis adults and their improved oviposition model
Source: PLoS One. 2020 Jul 15;15(7):e0235910. doi: 10.1371/journal.pone.0235910 (PMC7363081; doi:10.1371/journal.pone.0235910)
Supplement: S7 Table — (DOCX) [file pone.0235910.s007.docx]

**S7 Table. The estimated total fecundity of *Bactrocera dorsalis* at various constant temperatures**

| Temperature | Estimated total fecundity |
| --- | --- |
| 16.5 | 64.7111925 |
| 17 | 239.983689 |
| 17.5 | 405.5566166 |
| 18 | 561.4299753 |
| 18.5 | 707.6037652 |
| 19 | 844.0779861 |
| 19.5 | 970.8526382 |
| 20 | 1087.927721 |
| 20.5 | 1195.303236 |
| 21 | 1292.979181 |
| 21.5 | 1380.955558 |
| 22 | 1459.232365 |
| 22.5 | 1527.809604 |
| 23 | 1586.687274 |
| 23.5 | 1635.865375 |
| 24 | 1675.343907 |
| 24.5 | 1705.12287 |
| 25 | 1725.202265 |
| 25.5 | 1735.58209 |
| 26 | 1736.262347 |
| 26.5 | 1727.243034 |
| 27 | 1708.524153 |
| 27.5 | 1680.105703 |
| 28 | 1641.987684 |
| 28.5 | 1594.170096 |
| 29 | 1536.652939 |
| 29.5 | 1469.436214 |
| 30 | 1392.519919 |
| 30.5 | 1305.904056 |
| 31 | 1209.588624 |
| 31.5 | 1103.573622 |
| 32 | 987.8590524 |
| 32.5 | 862.4449134 |
| 33 | 727.3312056 |
| 33.5 | 582.5179289 |
| 34 | 428.0050833 |
| 34.5 | 263.7926688 |
| 35 | 89.88068547 |
| 35.2 | 17.60001284 |
