# Supplementary material for: Thermal effect on the fecundity and longevity of Bactrocera dorsalis adults and their improved oviposition model
Source: PLoS One. 2020 Jul 15;15(7):e0235910. doi: 10.1371/journal.pone.0235910 (PMC7363081; doi:10.1371/journal.pone.0235910)
Supplement: S8 Table — (DOCX) [file pone.0235910.s008.docx]

**S8 Table. The physiological age and cumulative proportion of egg production of *Bactrocera dorsalis* at various constant temperatures**

| Temperature | Physiological age | Cumulative proportion of egg production |
| --- | --- | --- |
| 20 | 0.111269615 | 0.000241109 |
|  | 0.119828816 | 0.007594937 |
|  | 0.128388017 | 0.021157324 |
|  | 0.136947218 | 0.037251356 |
|  | 0.145506419 | 0.066184448 |
|  | 0.154065621 | 0.094755877 |
|  | 0.162624822 | 0.136467752 |
|  | 0.171184023 | 0.164798071 |
|  | 0.179743224 | 0.197227245 |
|  | 0.188302425 | 0.241410488 |
|  | 0.196861626 | 0.275105485 |
|  | 0.205420827 | 0.306148282 |
|  | 0.213980029 | 0.339903556 |
|  | 0.22253923 | 0.375467149 |
|  | 0.231098431 | 0.403496082 |
|  | 0.239657632 | 0.43429777 |
|  | 0.248216833 | 0.456359253 |
|  | 0.256776034 | 0.480590717 |
|  | 0.265335235 | 0.50198915 |
|  | 0.273894437 | 0.52965642 |
|  | 0.282453638 | 0.555455093 |
|  | 0.291012839 | 0.57522604 |
|  | 0.29957204 | 0.604581073 |
|  | 0.308131241 | 0.622724533 |
|  | 0.316690442 | 0.638818565 |
|  | 0.325249643 | 0.652561784 |
|  | 0.333808845 | 0.671669681 |
|  | 0.342368046 | 0.690235081 |
|  | 0.350927247 | 0.702109705 |
|  | 0.359486448 | 0.716937914 |
|  | 0.368045649 | 0.725738397 |
|  | 0.37660485 | 0.735201929 |
|  | 0.385164051 | 0.741952984 |
|  | 0.393723252 | 0.753887884 |
|  | 0.402282454 | 0.764556962 |
|  | 0.410841655 | 0.772393008 |
|  | 0.419400856 | 0.782881254 |
|  | 0.427960057 | 0.790777577 |
|  | 0.436519258 | 0.797769741 |
|  | 0.445078459 | 0.803254973 |
|  | 0.45363766 | 0.809342978 |
|  | 0.462196862 | 0.813381555 |
|  | 0.470756063 | 0.824050633 |
|  | 0.479315264 | 0.831283906 |
|  | 0.487874465 | 0.838336347 |
|  | 0.496433666 | 0.844484629 |
|  | 0.504992867 | 0.856720916 |
|  | 0.513552068 | 0.864135021 |
|  | 0.52211127 | 0.869198312 |
|  | 0.530670471 | 0.872634117 |
|  | 0.539229672 | 0.875708258 |
|  | 0.547788873 | 0.880409885 |
|  | 0.556348074 | 0.883122363 |
|  | 0.564907275 | 0.885593731 |
|  | 0.573466476 | 0.888788427 |
|  | 0.582025678 | 0.892766727 |
|  | 0.590584879 | 0.896564195 |
|  | 0.59914408 | 0.903857746 |
|  | 0.607703281 | 0.909704641 |
|  | 0.616262482 | 0.911814346 |
|  | 0.624821683 | 0.918203737 |
|  | 0.633380884 | 0.921338156 |
|  | 0.641940086 | 0.923749247 |
|  | 0.650499287 | 0.923930078 |
|  | 0.659058488 | 0.924472574 |
|  | 0.667617689 | 0.925678119 |
|  | 0.67617689 | 0.927606992 |
|  | 0.684736091 | 0.93761302 |
|  | 0.693295292 | 0.938878843 |
|  | 0.701854494 | 0.940204943 |
|  | 0.710413695 | 0.941169379 |
|  | 0.718972896 | 0.942435202 |
|  | 0.727532097 | 0.944002411 |
|  | 0.736091298 | 0.944786016 |
|  | 0.744650499 | 0.947860157 |
|  | 0.7532097 | 0.948945148 |
|  | 0.761768902 | 0.949969861 |
|  | 0.770328103 | 0.950753466 |
|  | 0.778887304 | 0.951175407 |
|  | 0.787446505 | 0.953465943 |
|  | 0.796005706 | 0.955213984 |
|  | 0.804564907 | 0.955997589 |
|  | 0.813124108 | 0.95708258 |
|  | 0.82168331 | 0.958710066 |
|  | 0.830242511 | 0.963471971 |
|  | 0.838801712 | 0.966184448 |
|  | 0.847360913 | 0.967872212 |
|  | 0.855920114 | 0.968113321 |
|  | 0.864479315 | 0.972272453 |
|  | 0.873038516 | 0.975286317 |
|  | 0.881597718 | 0.975768535 |
|  | 0.89871612 | 0.97655214 |
|  | 0.907275321 | 0.978300181 |
|  | 0.915834522 | 0.978420735 |
|  | 0.924393723 | 0.979144063 |
|  | 0.932952924 | 0.980168776 |
|  | 0.941512126 | 0.983062086 |
|  | 0.950071327 | 0.983242917 |
|  | 0.958630528 | 0.983363472 |
|  | 0.967189729 | 0.984388186 |
|  | 0.97574893 | 0.985352622 |
|  | 0.984308131 | 0.986980108 |
|  | 0.992867332 | 0.987221218 |
|  | 1.001426534 | 0.987703436 |
|  | 1.009985735 | 0.989089813 |
|  | 1.018544936 | 0.989089813 |
|  | 1.027104137 | 0.990235081 |
|  | 1.035663338 | 0.991862568 |
|  | 1.044222539 | 0.992344786 |
|  | 1.05278174 | 0.9933695 |
|  | 1.061340942 | 0.993731163 |
|  | 1.069900143 | 0.994333936 |
|  | 1.087018545 | 0.995479204 |
|  | 1.095577746 | 0.996383363 |
|  | 1.104136947 | 0.997166968 |
|  | 1.112696148 | 0.997468354 |
|  | 1.12125535 | 0.997649186 |
|  | 1.129814551 | 0.998432791 |
|  | 1.138373752 | 0.998553345 |
|  | 1.146932953 | 0.998613623 |
|  | 1.155492154 | 0.9986739 |
|  | 1.164051355 | 0.999517782 |
|  | 1.172610556 | 0.999819168 |
|  | 1.181169757 | 1 |
| 24 | 0.101975781 | 0.003203572 |
|  | 0.114722753 | 0.0104197 |
|  | 0.127469726 | 0.038960619 |
|  | 0.140216699 | 0.086496457 |
|  | 0.152963671 | 0.129113678 |
|  | 0.165710644 | 0.174254927 |
|  | 0.178457616 | 0.222308514 |
|  | 0.191204589 | 0.272206582 |
|  | 0.203951562 | 0.315535709 |
|  | 0.216698534 | 0.367698929 |
|  | 0.229445507 | 0.410995696 |
|  | 0.242192479 | 0.457431317 |
|  | 0.254939452 | 0.494871048 |
|  | 0.267686424 | 0.534673009 |
|  | 0.280433397 | 0.570365337 |
|  | 0.29318037 | 0.597029415 |
|  | 0.305927342 | 0.63000356 |
|  | 0.318674315 | 0.653140472 |
|  | 0.331421287 | 0.678218943 |
|  | 0.34416826 | 0.699673171 |
|  | 0.356915233 | 0.71870045 |
|  | 0.369662205 | 0.735883248 |
|  | 0.382409178 | 0.757790506 |
|  | 0.39515615 | 0.769051548 |
|  | 0.407903123 | 0.784260428 |
|  | 0.420650096 | 0.796621687 |
|  | 0.433397068 | 0.810374397 |
|  | 0.446144041 | 0.817784681 |
|  | 0.458891013 | 0.832508171 |
|  | 0.471637986 | 0.840695078 |
|  | 0.484384959 | 0.849885124 |
|  | 0.497131931 | 0.854900819 |
|  | 0.509878904 | 0.863217163 |
|  | 0.522625876 | 0.869397793 |
|  | 0.535372849 | 0.874445847 |
|  | 0.548119822 | 0.880108727 |
|  | 0.560866794 | 0.884833188 |
|  | 0.573613767 | 0.891337411 |
|  | 0.586360739 | 0.895997152 |
|  | 0.599107712 | 0.900818691 |
|  | 0.611854685 | 0.909976378 |
|  | 0.624601657 | 0.915962852 |
|  | 0.63734863 | 0.92123742 |
|  | 0.650095602 | 0.924408633 |
|  | 0.662842575 | 0.928518267 |
|  | 0.675589547 | 0.932724978 |
|  | 0.68833652 | 0.935863832 |
|  | 0.701083493 | 0.939682231 |
|  | 0.713830465 | 0.941235479 |
|  | 0.726577438 | 0.945312753 |
|  | 0.73932441 | 0.946866 |
|  | 0.752071383 | 0.950587322 |
|  | 0.764818356 | 0.952625959 |
|  | 0.777565328 | 0.953305504 |
|  | 0.790312301 | 0.954179206 |
|  | 0.803059273 | 0.954826392 |
|  | 0.815806246 | 0.956444358 |
|  | 0.828553219 | 0.956767951 |
|  | 0.841300191 | 0.957932887 |
|  | 0.854047164 | 0.959227259 |
|  | 0.866794136 | 0.960489273 |
|  | 0.879541109 | 0.963854642 |
|  | 0.892288082 | 0.96586092 |
|  | 0.905035054 | 0.969420445 |
|  | 0.917782027 | 0.970908973 |
|  | 0.930528999 | 0.971912112 |
|  | 0.943275972 | 0.974209624 |
|  | 0.956022945 | 0.974565576 |
|  | 0.968769917 | 0.974921529 |
|  | 0.98151689 | 0.975536356 |
|  | 0.994263862 | 0.977477915 |
|  | 1.007010835 | 0.978804647 |
|  | 1.019757808 | 0.983529107 |
|  | 1.03250478 | 0.983755622 |
|  | 1.045251753 | 0.984079216 |
|  | 1.057998725 | 0.984855839 |
|  | 1.070745698 | 0.9857619 |
|  | 1.08349267 | 0.986926836 |
|  | 1.096239643 | 0.986959195 |
|  | 1.108986616 | 0.987315147 |
|  | 1.121733588 | 0.987379866 |
|  | 1.134480561 | 0.987541663 |
|  | 1.147227533 | 0.987703459 |
|  | 1.159974506 | 0.988188849 |
|  | 1.172721479 | 0.988544801 |
|  | 1.185468451 | 0.989256706 |
|  | 1.198215424 | 0.989903893 |
|  | 1.210962396 | 0.990421642 |
|  | 1.223709369 | 0.991068828 |
|  | 1.236456342 | 0.992589716 |
|  | 1.249203314 | 0.994175323 |
|  | 1.261950287 | 0.994563635 |
|  | 1.274697259 | 0.994984306 |
|  | 1.287444232 | 0.995793289 |
|  | 1.300191205 | 0.996505194 |
|  | 1.312938177 | 0.99666699 |
|  | 1.32568515 | 0.996731709 |
|  | 1.338432122 | 0.997249458 |
|  | 1.351179095 | 0.997411255 |
|  | 1.363926068 | 0.997411255 |
|  | 1.37667304 | 0.998705627 |
|  | 1.389420013 | 0.999191017 |
|  | 1.402166985 | 0.999482251 |
|  | 1.414913958 | 0.99954697 |
|  | 1.427660931 | 0.999805844 |
|  | 1.440407903 | 0.999870563 |
|  | 1.516889739 | 0.999935281 |
|  | 1.529636711 | 0.999967641 |
|  | 1.695347355 | 1 |
| 28 | 0.115606936 | 0.006087602 |
|  | 0.138728324 | 0.051744618 |
|  | 0.161849711 | 0.110876021 |
|  | 0.184971098 | 0.167260579 |
|  | 0.208092486 | 0.226132146 |
|  | 0.231213873 | 0.283964365 |
|  | 0.25433526 | 0.339420935 |
|  | 0.277456647 | 0.395879733 |
|  | 0.300578035 | 0.441648107 |
|  | 0.323699422 | 0.491314031 |
|  | 0.346820809 | 0.535523385 |
|  | 0.369942197 | 0.571640683 |
|  | 0.393063584 | 0.622383073 |
|  | 0.416184971 | 0.665850037 |
|  | 0.439306358 | 0.694617669 |
|  | 0.462427746 | 0.720601336 |
|  | 0.485549133 | 0.739420935 |
|  | 0.50867052 | 0.763400148 |
|  | 0.531791908 | 0.783964365 |
|  | 0.554913295 | 0.806644395 |
|  | 0.578034682 | 0.828990349 |
|  | 0.601156069 | 0.846325167 |
|  | 0.624277457 | 0.866592428 |
|  | 0.647398844 | 0.878470676 |
|  | 0.670520231 | 0.883890126 |
|  | 0.693641618 | 0.896362287 |
|  | 0.716763006 | 0.905419451 |
|  | 0.739884393 | 0.913808463 |
|  | 0.76300578 | 0.924795843 |
|  | 0.786127168 | 0.929992576 |
|  | 0.809248555 | 0.935857461 |
|  | 0.832369942 | 0.941462509 |
|  | 0.855491329 | 0.945471418 |
|  | 0.878612717 | 0.952449889 |
|  | 0.901734104 | 0.955753526 |
|  | 0.924855491 | 0.960690423 |
|  | 0.947976879 | 0.965070527 |
|  | 0.971098266 | 0.96796585 |
|  | 0.994219653 | 0.969153675 |
|  | 1.01734104 | 0.972865627 |
|  | 1.040462428 | 0.975946548 |
|  | 1.063583815 | 0.978619154 |
|  | 1.086705202 | 0.981069042 |
|  | 1.10982659 | 0.98359317 |
|  | 1.132947977 | 0.985115071 |
|  | 1.156069364 | 0.987861915 |
|  | 1.179190751 | 0.988455828 |
|  | 1.202312139 | 0.989903489 |
|  | 1.225433526 | 0.991239792 |
|  | 1.248554913 | 0.992613215 |
|  | 1.271676301 | 0.993058649 |
|  | 1.294797688 | 0.994729027 |
|  | 1.317919075 | 0.995694135 |
|  | 1.341040462 | 0.996585004 |
|  | 1.36416185 | 0.997512992 |
|  | 1.387283237 | 0.997698589 |
|  | 1.410404624 | 0.998515219 |
|  | 1.433526012 | 0.998886414 |
|  | 1.456647399 | 0.999331849 |
|  | 1.479768786 | 0.999480327 |
|  | 1.526011561 | 0.999554566 |
|  | 1.549132948 | 0.999628805 |
|  | 1.572254335 | 0.99996288 |
|  | 1.595375723 | 1 |
| 32 | 0.118715084 | 0.019771583 |
|  | 0.142458101 | 0.09216505 |
|  | 0.166201117 | 0.164804126 |
|  | 0.189944134 | 0.244750092 |
|  | 0.213687151 | 0.335257276 |
|  | 0.237430168 | 0.410536657 |
|  | 0.261173184 | 0.472491711 |
|  | 0.284916201 | 0.542551885 |
|  | 0.308659218 | 0.598857915 |
|  | 0.332402235 | 0.642883458 |
|  | 0.356145251 | 0.686540587 |
|  | 0.379888268 | 0.709259487 |
|  | 0.403631285 | 0.743153629 |
|  | 0.427374302 | 0.771337345 |
|  | 0.451117318 | 0.79620533 |
|  | 0.474860335 | 0.817266364 |
|  | 0.498603352 | 0.836423922 |
|  | 0.522346369 | 0.855274469 |
|  | 0.546089385 | 0.870318065 |
|  | 0.569832402 | 0.876642515 |
|  | 0.593575419 | 0.890826477 |
|  | 0.617318436 | 0.904150804 |
|  | 0.641061453 | 0.911826108 |
|  | 0.664804469 | 0.918396168 |
|  | 0.688547486 | 0.925334643 |
|  | 0.712290503 | 0.929878423 |
|  | 0.73603352 | 0.937430922 |
|  | 0.759776536 | 0.944553604 |
|  | 0.783519553 | 0.951553482 |
|  | 0.80726257 | 0.958798968 |
|  | 0.831005587 | 0.963711163 |
|  | 0.854748603 | 0.966904089 |
|  | 0.87849162 | 0.97071104 |
|  | 0.902234637 | 0.97525482 |
|  | 0.925977654 | 0.977465308 |
|  | 0.94972067 | 0.979614393 |
|  | 0.973463687 | 0.981763478 |
|  | 0.997206704 | 0.984219575 |
|  | 1.020949721 | 0.987965123 |
|  | 1.044692737 | 0.98888616 |
|  | 1.068435754 | 0.992140489 |
|  | 1.092178771 | 0.994105367 |
|  | 1.115921788 | 0.996500061 |
|  | 1.139664804 | 0.997789512 |
|  | 1.163407821 | 0.998894756 |
|  | 1.187150838 | 0.998894756 |
|  | 1.210893855 | 0.999447378 |
|  | 1.234636872 | 0.999877195 |
|  | 1.258379888 | 1 |
| 35 | 0.223097113 | 0.018811458 |
|  | 0.267716535 | 0.081658829 |
|  | 0.312335958 | 0.255237281 |
|  | 0.356955381 | 0.36425823 |
|  | 0.401574803 | 0.542539547 |
|  | 0.446194226 | 0.667379222 |
|  | 0.490813648 | 0.732791791 |
|  | 0.535433071 | 0.804617358 |
|  | 0.580052493 | 0.850790936 |
|  | 0.624671916 | 0.890979051 |
|  | 0.669291339 | 0.939290295 |
|  | 0.713910761 | 0.962377084 |
|  | 0.758530184 | 0.972210346 |
|  | 0.803149606 | 0.982043608 |
|  | 0.847769029 | 0.985891407 |
|  | 0.892388451 | 0.988884139 |
|  | 0.937007874 | 0.990166738 |
|  | 0.981627297 | 0.99187687 |
|  | 1.026246719 | 0.996152202 |
|  | 1.070866142 | 0.996579735 |
|  | 1.160104987 | 0.998717401 |
|  | 1.204724409 | 0.999144934 |
|  | 1.3832021 | 0.999572467 |
|  | 1.472440945 | 1 |
