# Supplementary material for: Thermal effect on the fecundity and longevity of Bactrocera dorsalis adults and their improved oviposition model
Source: PLoS One. 2020 Jul 15;15(7):e0235910. doi: 10.1371/journal.pone.0235910 (PMC7363081; doi:10.1371/journal.pone.0235910)
Supplement: S9 Table — (DOCX) [file pone.0235910.s009.docx]

**S9 Table. The estimated cumulative proportion of egg production of *Bactrocera dorsalis***

| Physiological age | Estimated proportion of egg production |
| --- | --- |
| 0 | - |
| 0.09 | - |
| 0.1 | - |
| 0.15 | 0.128826843 |
| 0.2 | 0.27764587 |
| 0.25 | 0.41366744 |
| 0.3 | 0.531178015 |
| 0.35 | 0.629551861 |
| 0.4 | 0.710158756 |
| 0.45 | 0.7751484 |
| 0.5 | 0.826871743 |
| 0.55 | 0.867593524 |
| 0.6 | 0.899356712 |
| 0.65 | 0.923930399 |
| 0.7 | 0.942803611 |
| 0.75 | 0.957203307 |
| 0.8 | 0.968123735 |
| 0.85 | 0.976359608 |
| 0.9 | 0.982538865 |
| 0.95 | 0.987152757 |
| 1 | 0.990582261 |
| 1.05 | 0.993120542 |
| 1.1 | 0.99499162 |
| 1.15 | 0.996365583 |
| 1.2 | 0.997370819 |
| 1.25 | 0.998103714 |
| 1.3 | 0.998636265 |
| 1.35 | 0.999021994 |
| 1.4 | 0.999300517 |
| 1.45 | 0.999501032 |
| 1.5 | 0.999644972 |
| 1.55 | 0.999748013 |
| 1.6 | 0.999821577 |
| 1.65 | 0.999873961 |
| 1.7 | 0.999911168 |
| 1.75 | 0.99993753 |
| 1.8 | 0.999956164 |
| 1.85 | 0.999969305 |
| 1.9 | 0.999978551 |
| 1.95 | 0.999985042 |
| 2 | 0.99998959 |
| 2.05 | 0.999992769 |
| 2.1 | 0.999994986 |
| 2.15 | 0.99999653 |
| 2.2 | 0.999997603 |
| 2.25 | 0.999998347 |
| 2.3 | 0.999998862 |
| 2.35 | 0.999999218 |
| 2.4 | 0.999999464 |
| 2.45 | 0.999999633 |
| 2.5 | 0.999999749 |
| 2.55 | 0.999999829 |
