# Supplementary material for: Thermal effect on the fecundity and longevity of Bactrocera dorsalis adults and their improved oviposition model
Source: PLoS One. 2020 Jul 15;15(7):e0235910. doi: 10.1371/journal.pone.0235910 (PMC7363081; doi:10.1371/journal.pone.0235910)
Supplement: S10 Table — (DOCX) [file pone.0235910.s010.docx]

**S10 Table. The estimated development rate of *Bactrocera dorsalis* female in the pre-oviposition at various constant temperatures**

| Temperature | Nonlinear function | Linear function |
| --- | --- | --- |
| 10 | -0.01927 | -0.05427 |
| 10.5 | -0.01711 | -0.04795 |
| 11 | -0.01468 | -0.04164 |
| 11.5 | -0.01198 | -0.03532 |
| 12 | -0.00903 | -0.029 |
| 12.5 | -0.00581 | -0.02268 |
| 13 | -0.00235 | -0.01637 |
| 13.5 | 0.001361 | -0.01005 |
| 14 | 0.005311 | -0.00373 |
| 14.5 | 0.009497 | 0.002584 |
| 15 | 0.013913 | 0.008901 |
| 15.5 | 0.018552 | 0.015218 |
| 16 | 0.023409 | 0.021535 |
| 16.5 | 0.028476 | 0.027852 |
| 17 | 0.033747 | 0.034169 |
| 17.5 | 0.039215 | 0.040485 |
| 18 | 0.044871 | 0.046802 |
| 18.5 | 0.050709 | 0.053119 |
| 19 | 0.05672 | 0.059436 |
| 19.5 | 0.062894 | 0.065753 |
| 20 | 0.069222 | 0.07207 |
| 20.5 | 0.075695 | 0.078387 |
| 21 | 0.082301 | 0.084704 |
| 21.5 | 0.089029 | 0.091021 |
| 22 | 0.095867 | 0.097338 |
| 22.5 | 0.102801 | 0.103655 |
| 23 | 0.109817 | 0.109972 |
| 23.5 | 0.1169 | 0.116289 |
| 24 | 0.124032 | 0.122606 |
| 24.5 | 0.131196 | 0.128923 |
| 25 | 0.13837 | 0.13524 |
| 25.5 | 0.145532 | 0.141557 |
| 26 | 0.152657 | 0.147874 |
| 26.5 | 0.159717 | 0.154191 |
| 27 | 0.16668 | 0.160508 |
| 27.5 | 0.17351 | 0.166825 |
| 28 | 0.180165 | 0.173142 |
| 28.5 | 0.186596 | 0.179459 |
| 29 | 0.192746 | 0.185776 |
| 29.5 | 0.198547 | 0.192093 |
| 30 | 0.203916 | 0.19841 |
| 30.5 | 0.208748 | 0.204727 |
| 31 | 0.212912 | 0.211044 |
| 31.5 | 0.216237 | 0.217361 |
| 32 | 0.218487 | 0.223678 |
| 32.5 | 0.219329 | 0.229995 |
| 33 | 0.218258 | 0.236311 |
| 33.5 | 0.214435 | 0.242628 |
| 34 | 0.206267 | 0.248945 |
| 34.5 | 0.189927 | 0.255262 |
| 35 | 0.148675 | 0.261579 |
| 35.05 | 0.139781 | 0.262211 |
