# Supplementary material for: Thermal effect on the fecundity and longevity of Bactrocera dorsalis adults and their improved oviposition model
Source: PLoS One. 2020 Jul 15;15(7):e0235910. doi: 10.1371/journal.pone.0235910 (PMC7363081; doi:10.1371/journal.pone.0235910)
Supplement: S11 Table — (DOCX) [file pone.0235910.s011.docx]

**S11 Table. The cumulative proportion for completing pre-ovipostion of *Bactrocera dorsalis* at various constant temperatures**

| Temperature | Physiological age | Cumulative proportion |
| --- | --- | --- |
| 16 | 0.603818616 | 0 |
|  | 0.630071599 | 0.090909091 |
|  | 0.708830549 | 0.181818182 |
|  | 0.735083532 | 0.272727273 |
|  | 0.866348449 | 0.454545455 |
|  | 0.892601432 | 0.454545455 |
|  | 0.971360382 | 0.545454545 |
|  | 1.102625298 | 0.636363636 |
|  | 1.155131265 | 0.727272727 |
|  | 1.207637232 | 0.818181818 |
|  | 1.286396181 | 0.909090909 |
|  | 1.470167064 | 1 |
| 20 | 0.68 | 0 |
|  | 0.741818182 | 0.058823529 |
|  | 0.803636364 | 0.176470588 |
|  | 0.865454545 | 0.294117647 |
|  | 0.927272727 | 0.588235294 |
|  | 1.050909091 | 0.705882353 |
|  | 1.112727273 | 0.823529412 |
|  | 1.174545455 | 0.882352941 |
|  | 1.298181818 | 0.941176471 |
|  | 1.483636364 | 1 |
| 24 | 0.670391061 | 0 |
|  | 0.782122905 | 0.1 |
|  | 0.893854749 | 0.25 |
|  | 1.005586592 | 0.7 |
|  | 1.117318436 | 1 |
| 28 | 0.551724138 | 0 |
|  | 0.735632184 | 0.3125 |
|  | 0.91954023 | 0.875 |
|  | 1.655172414 | 0.9375 |
|  | 1.83908046 | 0.9375 |
| 32 | 0.653846154 | 0 |
|  | 0.871794872 | 0.470588235 |
|  | 1.08974359 | 0.941176471 |
|  | 1.307692308 | 1 |
| 35 | 0.485294118 | 0 |
|  | 0.647058824 | 0.181818182 |
|  | 0.808823529 | 0.363636364 |
|  | 0.970588235 | 0.636363636 |
|  | 1.132352941 | 0.727272727 |
|  | 1.294117647 | 0.909090909 |
|  | 1.455882353 | 1 |
