# Supplementary material for: Thermal effect on the fecundity and longevity of Bactrocera dorsalis adults and their improved oviposition model
Source: PLoS One. 2020 Jul 15;15(7):e0235910. doi: 10.1371/journal.pone.0235910 (PMC7363081; doi:10.1371/journal.pone.0235910)
Supplement: S12 Table — (DOCX) [file pone.0235910.s012.docx]

**S12 Table. The estimated cumulative proportion of completing pre-oviposition period of *Bactrocera dorsalis* female**

| Physiological age | Estimated cumulative proportion |
| --- | --- |
| 0 | - |
| 0.1 | - |
| 0.2 | - |
| 0.3 | - |
| 0.45 | - |
| 0.5 | 0.000364498 |
| 0.55 | 0.011050011 |
| 0.6 | 0.04087552 |
| 0.65 | 0.091849059 |
| 0.7 | 0.162953351 |
| 0.75 | 0.2507502 |
| 0.8 | 0.349988807 |
| 0.85 | 0.454403639 |
| 0.9 | 0.557618206 |
| 0.95 | 0.653998007 |
| 1 | 0.739297332 |
| 1.05 | 0.81099704 |
| 1.1 | 0.868309941 |
| 1.15 | 0.911908719 |
| 1.2 | 0.94348444 |
| 1.25 | 0.965259171 |
| 1.3 | 0.979556709 |
| 1.35 | 0.988494143 |
| 1.4 | 0.993811586 |
| 1.45 | 0.996821802 |
| 1.5 | 0.998442652 |
| 1.55 | 0.999272443 |
| 1.6 | 0.999676175 |
| 1.65 | 0.999862783 |
| 1.7 | 0.999944682 |
| 1.75 | 0.999978797 |
| 1.8 | 0.999992278 |
| 1.85 | 0.99999733 |
| 1.9 | 0.999999124 |
| 1.95 | 0.999999727 |
| 2 | 0.99999992 |
