# Supplementary material for: Thermal effect on the fecundity and longevity of Bactrocera dorsalis adults and their improved oviposition model
Source: PLoS One. 2020 Jul 15;15(7):e0235910. doi: 10.1371/journal.pone.0235910 (PMC7363081; doi:10.1371/journal.pone.0235910)
Supplement: S13 Table — (DOCX) [file pone.0235910.s013.docx]

**S13 Table. The estimated development rate of *Bactrocera dorsalis* female in the oviposition at various constant temperatures**

| Temperature | Nonlinear function | Linear function |
| --- | --- | --- |
| 10 | 39.46334 | -0.0031 |
| 10.5 | 23.91296 | -0.00238 |
| 11 | 14.48425 | -0.00167 |
| 11.5 | 8.768177 | -0.00095 |
| 12 | 5.303627 | -0.00023 |
| 12.5 | 3.204438 | 0.000488 |
| 13 | 1.933156 | 0.001206 |
| 13.5 | 1.163832 | 0.001924 |
| 14 | 0.698791 | 0.002641 |
| 14.5 | 0.418158 | 0.003359 |
| 15 | 0.249244 | 0.004077 |
| 15.5 | 0.147976 | 0.004795 |
| 16 | 0.087634 | 0.005513 |
| 16.5 | 0.052026 | 0.006231 |
| 17 | 0.031337 | 0.006948 |
| 17.5 | 0.019626 | 0.007666 |
| 18 | 0.013294 | 0.008384 |
| 18.5 | 0.010167 | 0.009102 |
| 19 | 0.008931 | 0.00982 |
| 19.5 | 0.008795 | 0.010537 |
| 20 | 0.009282 | 0.011255 |
| 20.5 | 0.010108 | 0.011973 |
| 21 | 0.011104 | 0.012691 |
| 21.5 | 0.01217 | 0.013409 |
| 22 | 0.013249 | 0.014126 |
| 22.5 | 0.014309 | 0.014844 |
| 23 | 0.015331 | 0.015562 |
| 23.5 | 0.016309 | 0.01628 |
| 24 | 0.017238 | 0.016998 |
| 24.5 | 0.018117 | 0.017715 |
| 25 | 0.018949 | 0.018433 |
| 25.5 | 0.019736 | 0.019151 |
| 26 | 0.02048 | 0.019869 |
| 26.5 | 0.021184 | 0.020587 |
| 27 | 0.021852 | 0.021304 |
| 27.5 | 0.022487 | 0.022022 |
| 28 | 0.023093 | 0.02274 |
| 28.5 | 0.023676 | 0.023458 |
| 29 | 0.024242 | 0.024176 |
| 29.5 | 0.024801 | 0.024893 |
| 30 | 0.025369 | 0.025611 |
| 30.5 | 0.025967 | 0.026329 |
| 31 | 0.026632 | 0.027047 |
| 31.5 | 0.027425 | 0.027765 |
| 32 | 0.028443 | 0.028482 |
| 32.5 | 0.029845 | 0.0292 |
| 33 | 0.031894 | 0.029918 |
| 33.5 | 0.03502 | 0.030636 |
| 34 | 0.039934 | 0.031354 |
| 34.5 | 0.047807 | 0.032071 |
| 35 | 0.060569 | 0.032789 |
| 35.05 | 0.062222 | 0.032861 |
| 36 | 0.115546 | 0.034225 |
| 36.5 | 0.171649 | 0.034943 |
| 37 | 0.263964 | 0.03566 |
| 37.5 | 0.41599 | 0.036378 |
| 38 | 0.666468 | 0.037096 |
| 38.5 | 1.079274 | 0.037814 |
| 39 | 1.75972 | 0.038532 |
| 39.5 | 2.881436 | 0.03925 |
| 40 | 4.730688 | 0.039967 |
