# Supplementary material for: Thermal effect on the fecundity and longevity of Bactrocera dorsalis adults and their improved oviposition model
Source: PLoS One. 2020 Jul 15;15(7):e0235910. doi: 10.1371/journal.pone.0235910 (PMC7363081; doi:10.1371/journal.pone.0235910)
Supplement: S14 Table — (DOCX) [file pone.0235910.s014.docx]

**S14 Table. The cumulative proportion of oviposition of *Bactrocera dorsalis* female at various constant temperatures**

| Temperature | Physiological age | Cumulative proportion |
| --- | --- | --- |
| 20 | 0.009697661 | 0.042224635 |
|  | 0.019395322 | 0.076607552 |
|  | 0.029092983 | 0.109542768 |
|  | 0.038790645 | 0.142719266 |
|  | 0.048488306 | 0.185667752 |
|  | 0.058185967 | 0.224152491 |
|  | 0.067883628 | 0.259379901 |
|  | 0.077581289 | 0.285378212 |
|  | 0.08727895 | 0.322053324 |
|  | 0.096976612 | 0.356737845 |
|  | 0.106674273 | 0.382917119 |
|  | 0.116371934 | 0.417119073 |
|  | 0.126069595 | 0.440342623 |
|  | 0.135767256 | 0.467969598 |
|  | 0.145464917 | 0.491253468 |
|  | 0.155162578 | 0.515683436 |
|  | 0.16486024 | 0.536433828 |
|  | 0.174557901 | 0.562975027 |
|  | 0.184255562 | 0.583242852 |
|  | 0.193953223 | 0.606888648 |
|  | 0.203650884 | 0.624864278 |
|  | 0.213348545 | 0.650681626 |
|  | 0.223046207 | 0.670044637 |
|  | 0.232743868 | 0.686753529 |
|  | 0.242441529 | 0.704487875 |
|  | 0.25213919 | 0.715586922 |
|  | 0.261836851 | 0.726324044 |
|  | 0.271534512 | 0.73935336 |
|  | 0.281232173 | 0.752201713 |
|  | 0.290929835 | 0.761853058 |
|  | 0.300627496 | 0.767402582 |
|  | 0.310325157 | 0.775968151 |
|  | 0.320022818 | 0.782362167 |
|  | 0.329720479 | 0.793762818 |
|  | 0.33941814 | 0.802449029 |
|  | 0.349115801 | 0.812824225 |
|  | 0.358813463 | 0.819097599 |
|  | 0.368511124 | 0.825612257 |
|  | 0.378208785 | 0.829352153 |
|  | 0.387906446 | 0.834961998 |
|  | 0.397604107 | 0.842381469 |
|  | 0.407301768 | 0.852997949 |
|  | 0.41699943 | 0.857341054 |
|  | 0.426697091 | 0.8669924 |
|  | 0.436394752 | 0.872602244 |
|  | 0.446092413 | 0.877367596 |
|  | 0.455790074 | 0.881590059 |
|  | 0.465487735 | 0.883158403 |
|  | 0.475185396 | 0.890879479 |
|  | 0.484883058 | 0.895282905 |
|  | 0.494580719 | 0.898479913 |
|  | 0.50427838 | 0.900108578 |
|  | 0.513976041 | 0.903003981 |
|  | 0.523673702 | 0.907407407 |
|  | 0.533371363 | 0.908975751 |
|  | 0.543069025 | 0.914766558 |
|  | 0.552766686 | 0.916334902 |
|  | 0.562464347 | 0.920678007 |
|  | 0.572162008 | 0.925805284 |
|  | 0.581859669 | 0.928338762 |
|  | 0.59155733 | 0.929786464 |
|  | 0.601254991 | 0.930992882 |
|  | 0.610952653 | 0.93286283 |
|  | 0.620650314 | 0.935697913 |
|  | 0.630347975 | 0.939618772 |
|  | 0.640045636 | 0.942634817 |
|  | 0.649743297 | 0.943660273 |
|  | 0.659440958 | 0.94414284 |
|  | 0.66913862 | 0.944987333 |
|  | 0.678836281 | 0.945530221 |
|  | 0.688533942 | 0.946977923 |
|  | 0.698231603 | 0.948546266 |
|  | 0.707929264 | 0.949089154 |
|  | 0.717626925 | 0.951140065 |
|  | 0.727324586 | 0.953070334 |
|  | 0.737022248 | 0.954940282 |
|  | 0.746719909 | 0.95608638 |
|  | 0.75641757 | 0.959584992 |
|  | 0.766115231 | 0.960248522 |
|  | 0.775812892 | 0.961877187 |
|  | 0.785510553 | 0.96344553 |
|  | 0.795208214 | 0.96477259 |
|  | 0.804905876 | 0.965677404 |
|  | 0.814603537 | 0.96809024 |
|  | 0.824301198 | 0.970563397 |
|  | 0.833998859 | 0.971468211 |
|  | 0.84369652 | 0.972433345 |
|  | 0.853394181 | 0.975147786 |
|  | 0.863091843 | 0.978344794 |
|  | 0.872789504 | 0.978827362 |
|  | 0.882487165 | 0.979128966 |
|  | 0.892184826 | 0.979551212 |
|  | 0.901882487 | 0.981240198 |
|  | 0.911580148 | 0.98136084 |
|  | 0.921277809 | 0.982627579 |
|  | 0.930975471 | 0.983894318 |
|  | 0.940673132 | 0.984859452 |
|  | 0.950370793 | 0.984980094 |
|  | 0.960068454 | 0.986186512 |
|  | 0.969766115 | 0.987332609 |
|  | 0.979463776 | 0.987694535 |
|  | 0.989161438 | 0.989021595 |
|  | 0.998859099 | 0.990408976 |
|  | 1.00855676 | 0.991072506 |
|  | 1.018254421 | 0.991253468 |
|  | 1.027952082 | 0.991675715 |
|  | 1.037649743 | 0.991856678 |
|  | 1.047347404 | 0.993244058 |
|  | 1.057045066 | 0.993786947 |
|  | 1.066742727 | 0.995114007 |
|  | 1.076440388 | 0.995294969 |
|  | 1.086138049 | 0.995475932 |
|  | 1.09583571 | 0.99601882 |
|  | 1.105533371 | 0.996802992 |
|  | 1.115231033 | 0.997587164 |
|  | 1.124928694 | 0.997949089 |
|  | 1.134626355 | 0.998431656 |
|  | 1.144324016 | 0.998552298 |
|  | 1.154021677 | 0.998552298 |
|  | 1.163719338 | 0.998612619 |
|  | 1.173416999 | 0.998733261 |
|  | 1.183114661 | 0.998853903 |
|  | 1.192812322 | 0.999758716 |
|  | 1.202509983 | 0.999939679 |
|  | 1.212207644 | 1 |
| 24 | 0.014388489 | 0.037569168 |
|  | 0.028776978 | 0.087078924 |
|  | 0.043165468 | 0.127398634 |
|  | 0.057553957 | 0.174125489 |
|  | 0.071942446 | 0.214736433 |
|  | 0.086330935 | 0.266155389 |
|  | 0.100719424 | 0.314014821 |
|  | 0.115107914 | 0.35863832 |
|  | 0.129496403 | 0.408212795 |
|  | 0.143884892 | 0.453612918 |
|  | 0.158273381 | 0.495388797 |
|  | 0.172661871 | 0.535546711 |
|  | 0.18705036 | 0.567744232 |
|  | 0.201438849 | 0.603015888 |
|  | 0.215827338 | 0.62903278 |
|  | 0.230215827 | 0.657055949 |
|  | 0.244604317 | 0.679707472 |
|  | 0.258992806 | 0.703782804 |
|  | 0.273381295 | 0.720997961 |
|  | 0.287769784 | 0.738925023 |
|  | 0.302158273 | 0.757563991 |
|  | 0.316546763 | 0.775232178 |
|  | 0.330935252 | 0.788078827 |
|  | 0.345323741 | 0.800504805 |
|  | 0.35971223 | 0.812024723 |
|  | 0.374100719 | 0.821894314 |
|  | 0.388489209 | 0.832702327 |
|  | 0.402877698 | 0.842604278 |
|  | 0.417266187 | 0.850532311 |
|  | 0.431654676 | 0.85671294 |
|  | 0.446043165 | 0.863929068 |
|  | 0.460431655 | 0.87001262 |
|  | 0.474820144 | 0.87525483 |
|  | 0.489208633 | 0.881241303 |
|  | 0.503597122 | 0.885965764 |
|  | 0.517985612 | 0.892243472 |
|  | 0.532374101 | 0.897032651 |
|  | 0.54676259 | 0.90182183 |
|  | 0.561151079 | 0.908843802 |
|  | 0.575539568 | 0.915056791 |
|  | 0.589928058 | 0.921010905 |
|  | 0.604316547 | 0.924149759 |
|  | 0.618705036 | 0.928971297 |
|  | 0.633093525 | 0.932757337 |
|  | 0.647482014 | 0.937061127 |
|  | 0.661870504 | 0.939002686 |
|  | 0.676258993 | 0.941106041 |
|  | 0.690647482 | 0.943986021 |
|  | 0.705035971 | 0.947027797 |
|  | 0.71942446 | 0.950490244 |
|  | 0.73381295 | 0.952593599 |
|  | 0.748201439 | 0.953208426 |
|  | 0.762589928 | 0.954341002 |
|  | 0.776978417 | 0.955279423 |
|  | 0.791366906 | 0.955958968 |
|  | 0.805755396 | 0.95637964 |
|  | 0.820143885 | 0.958547714 |
|  | 0.834532374 | 0.959745009 |
|  | 0.848920863 | 0.961718927 |
|  | 0.863309353 | 0.964016439 |
|  | 0.877697842 | 0.966281591 |
|  | 0.892086331 | 0.969517523 |
|  | 0.90647482 | 0.970779536 |
|  | 0.920863309 | 0.972526939 |
|  | 0.935251799 | 0.974144905 |
|  | 0.949640288 | 0.974630295 |
|  | 0.964028777 | 0.974889169 |
|  | 0.978417266 | 0.975180403 |
|  | 0.992805755 | 0.976960166 |
|  | 1.007194245 | 0.979937223 |
|  | 1.021582734 | 0.982493609 |
|  | 1.035971223 | 0.982784843 |
|  | 1.050359712 | 0.984079216 |
|  | 1.064748201 | 0.984920558 |
|  | 1.079136691 | 0.985729541 |
|  | 1.09352518 | 0.986926836 |
|  | 1.107913669 | 0.986959195 |
|  | 1.122302158 | 0.987315147 |
|  | 1.136690647 | 0.987347507 |
|  | 1.151079137 | 0.987541663 |
|  | 1.165467626 | 0.987703459 |
|  | 1.179856115 | 0.988221208 |
|  | 1.194244604 | 0.988900754 |
|  | 1.208633094 | 0.989159629 |
|  | 1.223021583 | 0.989774456 |
|  | 1.237410072 | 0.990421642 |
|  | 1.251798561 | 0.991036469 |
|  | 1.26618705 | 0.992460279 |
|  | 1.28057554 | 0.994013526 |
|  | 1.294964029 | 0.994531275 |
|  | 1.309352518 | 0.994951946 |
|  | 1.323741007 | 0.995599133 |
|  | 1.338129496 | 0.996343397 |
|  | 1.352517986 | 0.996634631 |
|  | 1.366906475 | 0.996731709 |
|  | 1.381294964 | 0.997249458 |
|  | 1.395683453 | 0.997411255 |
|  | 1.410071942 | 0.997411255 |
|  | 1.424460432 | 0.998705627 |
|  | 1.438848921 | 0.999191017 |
|  | 1.45323741 | 0.999482251 |
|  | 1.467625899 | 0.99954697 |
|  | 1.482014388 | 0.999805844 |
|  | 1.496402878 | 0.999870563 |
|  | 1.510791367 | 0.999870563 |
|  | 1.525179856 | 0.999870563 |
|  | 1.539568345 | 0.999870563 |
|  | 1.553956835 | 0.999870563 |
|  | 1.568345324 | 0.999870563 |
|  | 1.582733813 | 0.999935281 |
|  | 1.597122302 | 0.999967641 |
|  | 1.611510791 | 0.999967641 |
|  | 1.625899281 | 0.999967641 |
|  | 1.64028777 | 0.999967641 |
|  | 1.654676259 | 0.999967641 |
|  | 1.669064748 | 0.999967641 |
|  | 1.683453237 | 0.999967641 |
|  | 1.697841727 | 0.999967641 |
|  | 1.712230216 | 0.999967641 |
|  | 1.726618705 | 0.999967641 |
|  | 1.741007194 | 0.999967641 |
|  | 1.755395683 | 0.999967641 |
|  | 1.769784173 | 0.999967641 |
|  | 1.784172662 | 0.999967641 |
|  | 1.798561151 | 0.999967641 |
|  | 1.81294964 | 1 |
| 28 | 0.026446281 | 0.041948177 |
|  | 0.052892562 | 0.101640805 |
|  | 0.079338843 | 0.166493429 |
|  | 0.105785124 | 0.232274111 |
|  | 0.132231405 | 0.292115228 |
|  | 0.158677686 | 0.346907714 |
|  | 0.185123967 | 0.40396466 |
|  | 0.211570248 | 0.454673695 |
|  | 0.238016529 | 0.501447769 |
|  | 0.26446281 | 0.546996807 |
|  | 0.290909091 | 0.581000817 |
|  | 0.317355372 | 0.626252877 |
|  | 0.343801653 | 0.669945801 |
|  | 0.370247934 | 0.698864058 |
|  | 0.396694215 | 0.726928502 |
|  | 0.423140496 | 0.747271512 |
|  | 0.449586777 | 0.76969337 |
|  | 0.476033058 | 0.790259114 |
|  | 0.502479339 | 0.811975648 |
|  | 0.52892562 | 0.831204989 |
|  | 0.555371901 | 0.849023684 |
|  | 0.581818182 | 0.867139357 |
|  | 0.608264463 | 0.880651867 |
|  | 0.634710744 | 0.885886109 |
|  | 0.661157025 | 0.895946247 |
|  | 0.687603306 | 0.905078328 |
|  | 0.714049587 | 0.912688396 |
|  | 0.740495868 | 0.923379612 |
|  | 0.766942149 | 0.928762343 |
|  | 0.79338843 | 0.935555721 |
|  | 0.819834711 | 0.941309674 |
|  | 0.846280992 | 0.946952261 |
|  | 0.872727273 | 0.950775856 |
|  | 0.899173554 | 0.956492687 |
|  | 0.925619835 | 0.95931398 |
|  | 0.952066116 | 0.964139877 |
|  | 0.978512397 | 0.967963472 |
|  | 1.004958678 | 0.970302175 |
|  | 1.031404959 | 0.972640879 |
|  | 1.05785124 | 0.974645482 |
|  | 1.084297521 | 0.978469077 |
|  | 1.110743802 | 0.980176702 |
|  | 1.137190083 | 0.983294974 |
|  | 1.163636364 | 0.984631376 |
|  | 1.190082645 | 0.986598857 |
|  | 1.216528926 | 0.98812087 |
|  | 1.242975207 | 0.988529215 |
|  | 1.269421488 | 0.99105353 |
|  | 1.295867769 | 0.991424753 |
|  | 1.32231405 | 0.992612666 |
|  | 1.348760331 | 0.993095256 |
|  | 1.375206612 | 0.994728636 |
|  | 1.401652893 | 0.99576806 |
|  | 1.428099174 | 0.99658475 |
|  | 1.454545455 | 0.997549929 |
|  | 1.480991736 | 0.997809785 |
|  | 1.507438017 | 0.998515109 |
|  | 1.533884298 | 0.998886332 |
|  | 1.560330579 | 0.999331799 |
|  | 1.58677686 | 0.999480288 |
|  | 1.61322314 | 0.999480288 |
|  | 1.639669421 | 0.999554533 |
|  | 1.666115702 | 0.999628777 |
|  | 1.692561983 | 0.999962878 |
|  | 1.719008264 | 1 |
| 32 | 0.026645768 | 0.051639445 |
|  | 0.053291536 | 0.130111752 |
|  | 0.079937304 | 0.201829792 |
|  | 0.106583072 | 0.285644112 |
|  | 0.13322884 | 0.376396905 |
|  | 0.159874608 | 0.44645708 |
|  | 0.186520376 | 0.512034877 |
|  | 0.213166144 | 0.577305661 |
|  | 0.239811912 | 0.629559131 |
|  | 0.26645768 | 0.668242662 |
|  | 0.293103448 | 0.696057964 |
|  | 0.319749216 | 0.733145033 |
|  | 0.346394984 | 0.758381432 |
|  | 0.373040752 | 0.785951124 |
|  | 0.39968652 | 0.810020877 |
|  | 0.426332288 | 0.827397765 |
|  | 0.452978056 | 0.850362274 |
|  | 0.479623824 | 0.86061648 |
|  | 0.506269592 | 0.873879406 |
|  | 0.532915361 | 0.881370502 |
|  | 0.559561129 | 0.895124647 |
|  | 0.586206897 | 0.907159524 |
|  | 0.612852665 | 0.913054157 |
|  | 0.639498433 | 0.920790863 |
|  | 0.666144201 | 0.927545131 |
|  | 0.692789969 | 0.931167874 |
|  | 0.719435737 | 0.938290556 |
|  | 0.746081505 | 0.946088665 |
|  | 0.772727273 | 0.954746408 |
|  | 0.799373041 | 0.95984281 |
|  | 0.826018809 | 0.964202382 |
|  | 0.852664577 | 0.967640919 |
|  | 0.879310345 | 0.973596954 |
|  | 0.905956113 | 0.975684637 |
|  | 0.932601881 | 0.978324942 |
|  | 0.959247649 | 0.980351222 |
|  | 0.985893417 | 0.98182488 |
|  | 1.012539185 | 0.986675672 |
|  | 1.039184953 | 0.988210733 |
|  | 1.065830721 | 0.990544026 |
|  | 1.092476489 | 0.992877318 |
|  | 1.119122257 | 0.995087805 |
|  | 1.145768025 | 0.996622866 |
|  | 1.172413793 | 0.998526342 |
|  | 1.199059561 | 0.998894756 |
|  | 1.225705329 | 0.999263171 |
|  | 1.252351097 | 0.999692988 |
|  | 1.278996865 | 1 |
| 35 | 0.057591623 | 0.156303972 |
|  | 0.115183246 | 0.40716753 |
|  | 0.172774869 | 0.537564767 |
|  | 0.230366492 | 0.621761658 |
|  | 0.287958115 | 0.700777202 |
|  | 0.345549738 | 0.769861831 |
|  | 0.403141361 | 0.822970639 |
|  | 0.460732984 | 0.900690846 |
|  | 0.518324607 | 0.920120898 |
|  | 0.57591623 | 0.95164076 |
|  | 0.633507853 | 0.966321244 |
|  | 0.691099476 | 0.979274611 |
|  | 0.748691099 | 0.980569948 |
|  | 0.806282723 | 0.988773748 |
|  | 0.863874346 | 0.991364421 |
|  | 0.921465969 | 0.991364421 |
|  | 0.979057592 | 0.994818653 |
|  | 1.036649215 | 0.99611399 |
|  | 1.094240838 | 0.998272884 |
|  | 1.151832461 | 0.998272884 |
|  | 1.209424084 | 0.998272884 |
|  | 1.267015707 | 0.999136442 |
|  | 1.32460733 | 0.999568221 |
|  | 1.382198953 | 0.999568221 |
|  | 1.439790576 | 0.999568221 |
|  | 1.497382199 | 0.999568221 |
|  | 1.554973822 | 1 |
