# Supplementary material for: Thermal effect on the fecundity and longevity of Bactrocera dorsalis adults and their improved oviposition model
Source: PLoS One. 2020 Jul 15;15(7):e0235910. doi: 10.1371/journal.pone.0235910 (PMC7363081; doi:10.1371/journal.pone.0235910)
Supplement: S15 Table — (DOCX) [file pone.0235910.s015.docx]

**S15 Table. The estimated cumulative proportion of oviposition of *Bactrocera dorsalis* female**

| Physiological age | Estimated proportion |
| --- | --- |
| 0 | 0 |
| 0.1 | 0.329693903 |
| 0.2 | 0.562169824 |
| 0.3 | 0.716962649 |
| 0.4 | 0.818279978 |
| 0.5 | 0.883931109 |
| 0.6 | 0.926172164 |
| 0.7 | 0.953204141 |
| 0.8 | 0.970427592 |
| 0.9 | 0.981361315 |
| 1 | 0.988280314 |
| 1.1 | 0.992646612 |
| 1.2 | 0.995395201 |
| 1.3 | 0.997121584 |
| 1.4 | 0.998203723 |
| 1.5 | 0.99888077 |
| 1.6 | 0.999303638 |
| 1.7 | 0.999567329 |
| 1.8 | 0.999731514 |
| 1.9 | 0.999833599 |
| 2 | 0.999896989 |
| 2.1 | 0.9999363 |
| 2.2 | 0.999960651 |
| 2.3 | 0.999975718 |
| 2.4 | 0.99998503 |
| 2.5 | 0.999990779 |
| 2.6 | 0.999994326 |
| 2.7 | 0.999996511 |
| 2.8 | 0.999997857 |
| 2.9 | 0.999998684 |
| 3 | 0.999999193 |
| 3.1 | 0.999999505 |
| 3.2 | 0.999999697 |
