# Supplementary material for: Thermal effect on the fecundity and longevity of Bactrocera dorsalis adults and their improved oviposition model
Source: PLoS One. 2020 Jul 15;15(7):e0235910. doi: 10.1371/journal.pone.0235910 (PMC7363081; doi:10.1371/journal.pone.0235910)

Manuscript Click here to access/download;Manuscript;texts_0201.docx

Submitted to PLOS ONE

Short title: Oviposition model of *B. dorsalis*

Thermal effect on the fecundity and longevity of *Bactrocera dorsalis* adults and their improved oviposition model

Kyung San Choi[1], Ana Clariza Samayoa[2], Shaw-Yhi Hwang[2], Yu-Bing Huang[3], Jeong Joon Ahn[1*]

[1] Research Institute of Climate Change and Agriculture, National Institute of Horticultural and Herbal science, Rural Development Administration, Jeju 63240, Republic of Korea

[2] Department of Entomology, National Chung Hsing University, Taichung, 40227, Taiwan (R.O.C)

[3] Taiwan Agricultural Research Institute, Taichung, 10617, Taiwan (R.O.C)

[*]Corresponding author: Jeong Joon Ahn

Research Institute of Climate Change and Agriculture, NIHHS, RDA, 281 Ayeonno, Jeju, 63240, Republic of Korea

E-mail: j2ahn33@korea.kr

Abstract

The oriental fruit fly, *Bactrocera dorsalis,* is a destructive polyphagous pest that causes damage to various fruit crops, and their distribution is currently expanding worldwide. Temperature is an important abiotic factor that influences insect population dynamics and distribution by affecting their survival, development, and reproduction. We examined the fecundity, pre-oviposition and oviposition periods, and longevity of adult *B. dorsalis* at various constant temperatures ranging from13ºC to 35ºC. The longevity of female *B. dorsalis* ranged from 116.8 days (18.8ºC) to 22.4 days (34.9ºC), and the maximum fecundity per female was 1,684 eggs at 28.1ºC. Females were only able to lay eggs at 16.7ºC to 34.9ºC, and both the pre-oviposition and oviposition periods were different depending on the temperature. We modeled female reproduction in two oviposition models (OMs): 1) the current model, an OM composed of a fecundity model, age-specific survival model, and age-specific cumulative oviposition rate model, and 2) a two-phase OM, separating pre-oviposition from the oviposition phase (OP) where oviposition was estimated for the females who had completed the pre-oviposition phase (POP). The results of the two-phase OM provided more realistic outputs at lower and higher temperatures than those of the current model. We discussed the usefulness of the two-phase OM for the reproduction of insects with long pre-oviposition periods.

.

Introduction

The oriental fruit fly *Bactrocera dorsalis* (Hendel) (Diptera: Tephritidae) is a destructive polyphagous pest that damages various fruits by piercing the skin for oviposition and consequent fruit destruction caused by hatched larva. Therefore, *B. dorsalis* also cause economic damage to the food industry due to quarantine issue [1-2]. Their distribution has rapidly expanded from their location of origin in South Asia to northern and eastern nations and islands, including Taiwan, Japan, and the Pacific Islands in the 1900s [2, 3-6]. In Taiwan, *B. dorsalis* became a major pest with annual occurrences throughout the state since its initial discovery in 1911 [7-8]. Area-wide management by annihilating the males of the species with effective methyl-eugenol attractants and insecticides reduced fruit damage and loss in several crops [9-10]. Japan succeeded in eradicating *B. dorsalis* in the 1980s [11], but subsequently faced persistent invasion from neighboring nations [12]. In China, although *B. dorsalis* has always been a dominant species [13], its distribution has continually expanded to mid-china below the overwintering limitation altitude of 30°N [14-16]. Although *B. dorsalis* adults have long-distance flight ability, enabling them to forage as far as 24 miles away [17], their settlement is greatly limited by the temperature and available host plants. *B. dorsalis* normally develop and survive in a temperature range of 15 ºC to 35ºC, unless they experience mass mortality and are unable to develop, inferring the studies on relationship between temperature and development and survival [14, 18-25]. Several studies have investigated the reproduction of *B. dorsalis* in relation to altering temperatures, food resources, dietary restrictions, polyandry, and glutamine synthetase [1, 26-31]. However, there have been fewer studies regarding oviposition modeling compared to modeling the immature stage's development. Several approaches exist for constructing oviposition models (OMs) for many arthropod pests [32-34] based on the logical structure developed by Kim and Le[illegible]5].

In this study, we investigated the effect of temperature on the fecundity and longevity of *B. dorsalis* adults and developed an improved model to precisely describe the relationship between temperature and oviposition. The modified model will contribute towards predictions of the seasonal occurrence and oviposition of *B. dorsalis*.

Materials and Methods

Insect colony

Bactrocera dorsalis pupae were provided by the Taiwan Agricultural Research Institute (TARI) and reared according to the method of Huang and Chi [29] based on an artificial diet developed by Tanaka et al. [36]. Pupae were placed in a plastic netted cage (30 x 30 cm) and emerged as adults, who were then fed an artificial diet composed of 200 g Yeast Hydrolysate Enzymatic (MP Biomedicals, LLC., Illkirch-Graffenstaden, France), 40 g granulated sugar, 10 ml protein hydrolysate (Alco Standard Co., Pennsylvania, USA), and 50 ml water. For the oviposition site, a plastic cylinder (4.5 cm diameter and 5 cm height) containing a 20% guava juice cotton ball was placed into the cage. Eggs were laid inside the cylinder and were collected using distilled water. Subsequently, approximately 4 mL of eggs were inoculated into a container (90 x 15 mm) filled with an artificial diet composed of 5 g sodium benzoate (Sigma-Aldrich Co. Milan, Italy), 240 g granulated sugar (Taiwan Sugar corporation, Tainan, Taiwan), 140 g yeast (Vietnam - Taiwan Sugar Co., Thanh Hoa, Vietnam), 20 mL HCI (Sigma-Aldrich Co., Austria), 480 g wheat grain (purchased at Taichung), and 1100 ml of distilled water. Matured larvae left the container and pupated in the surrounding sawdust. Pupae were collected by sieving the sawdust.

Laboratory experiment

The fecundity and longevity of *B. dorsalis* adults were examined in growth chambers (Model A 414931206, Yuh Chuen Chiou Industry Co., Kaohsiung, Taiwan) set to seven constant temperatures; 13ºC, 16ºC, 20ºC, 24ºC, 28ºC, 32ºC, and 35ºC, with a 14: 10 h (light: dark) photoperiod. Temperature and humidity in the chamber were recorded at 1 h intervals by a data logger (HOBO, ONSET computer, Co., USA). The humidity range was 50~70% at all temperatures except the chamber set to 20ºC, where it was 19~30 %.

One newly emerged (<12 h) virgin female and two males were placed into a cage (10 × 15 × 10 cm) with a supply of the artificial diet and a 10% sugar- containing gel. A female ~~reproducing~~ with two males produces significantly more eggs than females with only one male [29]. A perforated plastic cup (diameter and height both 4 cm) was provided for the oviposition site with a cotton ball soaked in guava juice placed inside the cup. The number of eggs laid per female in the cup was recorded daily. The adult oviposition period (AOP) of each female was determined by the day from which the female first laid a significant number (>5) of eggs consistently, as many females did not lay the eggs consistently at 13.5ºC, 16.7ºC and 34.9ºC. Adult pre-oviposition period (APOP) was defined from adult emergence to AOP, and total pre-oviposition period (TPOP) was obtained by summing the APOP and immature development period examined in previous study [25].

An analysis of variance was conducted to determine the statistical differences in adult longevity, fecundity, APOP, AOP, and TPOP using SAS [37] after checking normality of the data by Skewness, Kurtosis, and Kolmogrov Smirov methods. Means were separated using Tukey's honest significance test (HSD; P = 0.05).

Low developmental threshold and thermal constant

Aging rates (1/mean days of longevity) of both males and females were plotted against

temperature, respectively (Fig 1A and 1B). A linear regression was conducted in the region where rates increased linearly (over 18.8°C). ~~Two parameters, a (slope) and b (intercept),~~ of the linear model ($Y = \mathrm{a}X + \mathrm{b}$) were estimated using the Table Curve 2D program [38], and were used to deduce the low development threshold (LDT) and thermal constant (TC) by solving –b/a and 1/a, respectively. A linear regression test was applied to the region showing a proportional relationship between temperature and development rates of ATOP, AOP and ATOP, respectively.

Non-linear model development

Physiological age. The physiological age (Px) was obtained by accumulating the rates computed with a development model [$D(T)$] as an input of temperature T_i°C from the starting day (0) to the nth day [39]. Development models in this study are adult aging models and development rate models for pre-oviposition and oviposition periods, respectively (Fig 4)

$$Px = \int_{t_0}^{t_n} D[T(t)]\ dt \approx \sum_{i=0}^{n} D(T_i)\ \Delta t \tag{1}$$

Aging model. The aging rates (1/mean days of longevity) of both male and female adults were stationary under 20°C but then increased exponentially. The obtained flat then increasing curve is similar to the aging rate curve previously obtained for *Ascotis selenaria* [40]. We applied the function to describe the aging rate curves of males and females, respectively:

$$A(T) = r_0 + exp\left[\frac{(T - T_H)}{c}\right] \tag{2}$$

where $A(T)$ is the aging rate at T °C with a minimum aging rate (r_0) and ≈1 at T_H°C.

Age-specific survival rate model. ~~A survival number of each~~ adult male and female for each day after emergence at each temperature was ~~translated to~~ ~~a~~ relative survival proportion to the total number of examined adults at each temperature. Adult age was expressed in days spent at the specified temperature, and was normalized as a physiological age by dividing the number of days by the mean longevity of the temperature, which finally formed the age-specific probability distribution (Fig 1C and 1D). A modified two-parameter Weibull equation [41] was applied to compute relative survival ($S(Px)$) at a physiological age (Px):

$$S(Px) = \exp\left[-(Px/\alpha)^{\beta}\right] \tag{3}$$

Parameter α indicates the physiological age of 50% of surviving individuals and β is a shape parameter of the curve.

Total fecundity model. The relationship between total fecundity per female and temperature was calculated by the equation proposed by Briere et al [44]. Eggs were laid between 16.7°C and 34.9°C with a specifically high amount at 24°C and 28°C, shaping a campaniform (Fig 2A):

$$F(T) = \alpha \cdot (T - T_L) \cdot (T_H - T) \tag{4}$$

where $F(T)$ is the total number of eggs that a female is capable of laying in her life-span at a temperature T°C with low (T_L) and high (T_H) temperature limits. α is the empirical constant of the equation.

Age-specific cumulative oviposition rate model (ORM). The number of eggs laid by the female in a day after adult emergence in all examined temperatures was translated by normalizing with the physiological age, as described in the survival model, into the age-specific cumulative oviposition probability distribution (Fig 2B). Three-parameter Weibull function [42-43] was applied:

$$E(Px) = 1 - \exp\left[-\left(\frac{Px-\gamma}{\eta}\right)^{\beta}\right] \quad (5)$$

where $E(Px)$ is the cumulative proportion of eggs laid by the female at a physiological age (Px). Parameter γ is a physiological age when the first egg appeared in this model, and η, and β are parameters of the equation.

Pre-oviposition development rate model (PDRM) and completion distribution model (PCDM). The pre-oviposition development rate of *B. dorsalis* increased linearly as temperature increased up to 32°C, then decreased at 34.9°C (Fig 3A). In the pre-oviposition phase (POP), the Briere 2 model [44] was applied to describe the relationship between temperature and development rate:

$$D_p(T) = \alpha \cdot T \cdot (T - T_L) \cdot (T_H - T)^{\frac{1}{m}} \quad (6)$$

where $D_p(T)$ is the development rate model of POP and m is the empirical constant of the equation.

The pre-oviposition period of the females at different temperatures was translated to the age-specific cumulative completion distribution as described in the survival model (Fig 3B). The

three-parameter Weibull function in eq. (5) was applied to PCDM [$C_p(Px)$] to predict the probability of the female completing POP at a physiological age (Px).

Oviposition development rate model (ODRM) and age-specific cumulative oviposition rate model of* oviposition phase (*OP*) *(OORM). The development rates of the oviposition period showed a slowly increasing region from 16.7°C to 32.0°C, and steeply increased at either the end of the low or high temperatures (Fig 3B). A model showing a curve of best fit was selected for ODRM [$D_o(T)$] that computes a development rate of a female in OP at a temperature T °C.

$$D_o(T) = a + b \cdot \exp(T) + c/T^2 + d \cdot \exp(-T) \quad (7)$$

Eggs laid by the female during OP were translated to the age-specific cumulative oviposition probability distribution (Fig 3D). The two-parameter Weibull function used in eq. (3) was applied to compute the cumulative proportion of eggs laid by a female at a physiological age (Px) in OP.

Model selection and parameter estimation. The Table Curve 2D program [38] was used to select a best-fit model for ODRM, but was mostly used for parameter estimation of the models through statistical analysis when its curve was best fit to corresponding observed or translated data.

Oviposition models and their simulation

Oviposition model (OM). Kim and Lee [35] developed an OM composed of a fecundity model and two age-specific models, ORM and female survival rate model, based on the physiological

age derived from the female aging model (Fig 4A). We used a modified OM where the female survival expression $[(S(Px_i) + S(Px_{i-1}))/2]$ of the OM expression was simplified by Choi and Kim [40] as:

$$F(T) \cdot [E(Px_i) - E(Px_{i-1})] \cdot S(Px_i) \quad (i \geq 1,\ Px_0 = 0) \tag{8}$$

The amount of eggs laid by a female cohort on the ith day after adult emergence is the product of a fecundity of the cohort at temperature T°C [$F(T)$], a proportion of eggs laid by the female at the ith day $[E(Px_i) - E(Px_{i-1})]$, and the survival proportion of the female at the ith day $[S(Px_i)]$. Px_{i-1} and Px_i are physiological ages obtained through the accumulation of the aging rates derived by the female aging model [$A(T)$] to the $i\text{-}1^{th}$ and i^{th} day, respectively.

Two-phase OM. Egg production was simulated only in OP with the female that completed POP (Fig 4B). The newly emerged females required a time (= pre-oviposition period) to develop reproductive organ and complete mating. A proportion of the female completing POP at the ith day after adult emergence was predicted by subtracting the cumulative proportion at the $i\text{-}1^{th}$ day [$C_p(Px_{i-1})$] from that at the ith day [$C_p(Px_i)$], whose physiological ages, Px_{i-1} and Px_i, were obtained by summating the developmental rates from PDRM [$D_p(T)$]. A proportion of the females entered OP the following day (i+1th) and simulated the daily proportion of eggs laid at the jth day after the onset of OP was calculated by subtracting a proportion of cumulative oviposition at the $j\text{-}1^{th}$ day [$E_o(Px_{j-1})$] from that at the jth day [$E_o(Px_j)$], whose physiological ages, Px_{j-1} and Px_j, were calculated by ODRM [$D_o(T)$]. Therefore, two-phase OM is expressed as:

$$F(T) \cdot \left[E_o\left(Px_j\right) - E_o\left(Px_{j-1}\right)\right] \cdot S\left(Px_i\right) \quad (j \geq 1, i \geq 1, Px_0 = 0) \qquad (9)$$

Model simulation and validation. Both OMs, current OM and two-phase OM, were simulated to predict the daily egg production sequence laid by one female at a constant temperature ranging from 10°C to 40°C by 1°C accrual. Daily observed egg production at each temperature was examined in a chamber to validate models. The mean temperature of each observation was used for input. Simulation outputs of both models and observed eggs were compared by transforming a cumulative graph and Pearson correlation test. The PopModel 1.5 program [45] was used to simulate models as described above (Fig 6).

Results

Temperature effect on adult longevity and fecundity

Temperature affects the longevity and fecundity of the *B. dorsalis* adult (Table 1). Longevity in the adult female decreased from 116.8 d at 18.8 ºC to 22.4 d at 34.9ºC as the temperature increased over 18.8 ºC, while it was likely stationary below 18.8ºC except for a temporal decrease at 16.7 ºC ($F_{6, 118}$ = 27.69, P < 0.0001). Male showed a similar response against temperature but decreased longevity except at 13.5ºC ($F_{6, 200}$ = 68.96, P<0.0001). Eggs were laid from 16.7ºC to 34.9ºC. No eggs were found nor a vigorous activity of adults observed at 13.5ºC, inferring no ~~even~~ mating behaviors. As temperature increased, fecundity increased sharply from 63 eggs at 16.7ºC to 1,684 eggs at 28.1ºC and then decreased rapidly ($F_{6, 118}$ = 55.57, P < 0.0001). Both the pre-oviposition and oviposition period of the females were different to the temperature (Table 2). APOP became shorter from 38.1 d at 16.7ºC to 4.6 d at 32.0ºC, and then increased at 6.2 d at 34.9ºC ($F_{5, 86}$ = 130.25, P < 0.0001) and TPOP showed a similar tendency to APOP ranging from 91.2 d at 16.7ºC to 21.5 d at 32.0ºC. However, AOP

increased rapidly from 103.1 d at 18.8ºC to 23.6 d at 16.7ºC and then decreased to 17.4 d at 34.9ºC ($F_{5, 86}$ = 32.09, P < 0.0001).

Low development threshold (LDT) and thermal constant (TC)

A linear regression test on increasing aging rates over 18.8ºC (Fig 1A and 1B) showed that both adult males and females had similar LDT near 16.0ºC, but the TC of females was slightly higher than that of the male's (Table 3; Male: $F_{1, 3}$ = 15.89, P < 0.0283, Female: $F_{1, 3}$ = 15.02, P < 0.0304). LDTs and TCs of APOP, AOP, and TPOP were estimated onto the developmental rates and showed a linear relationship against temperature (Fig 3A and 3B, Table 3). TC of AOP was nine times higher than that of APOP, although the LDT of AOP was approximately 2ºC lower (APOP: $F_{1, 3}$ = 417.30[illegible]P = 0.00026, AOP: $F_{1, 2}$ = 20.5065, P = 0.04547). The LDT of TPOP decreased to 10.6ºC, close to LDT of the immature stage (Table 3; $F_{1, 3}$ = 96.5145, P = 0.00224).

Non-linear model development

Adult aging and survival model. The exponential type equation (2) used for an aging rate model in a moth [40] drew curves of best fit on the aging rates of both adult males and females, respectively (Fig 1A). Parameters of both aging models were estimated to be very close to each other (Male: $F_{2, 3}$ = 205.76; P < 0.0001; r^2 = 0.993, Female: $F_{2, 3}$ = 23.57; P = 0.0146; r^2 = 0.940) (Table 4). The minimum aging rate (r_0) was ≈ 0.01 and maximum temperature (T_H) was 52.0°C when the aging rate became ≈ 1.0. Survival at different temperatures was normalized in a probability distribution based on physiological age (Fig 1C and 1D). A modified two-parameter Weibull equation was well-fitted to adult survival (Male: $F_{1, 147}$ = 3585.8; P < 0.0001; r^2 = 0.961, Female: $F_{1, 109}$ = 3624.5; P < 0.0001; r^2 = 0.971) (Table 4). Parameter α was estimated as 1.0665 in males and 1.0921 in females, implying a physiological age when 50% of individuals survive, respectively.

Fecundity model and ORM. A quadratic equation was matched to the campaniform of the fecundity between 16.7˚C and 34.9 ˚C (Fig 2A). Parameters, T_L and T_H, were estimated as 16.3˚C and 35.2˚C, respectively ($F_{2, 3}$ = 99.57; P = 0.0018; r^2 = 0.985) (Table 5). Daily egg production laid by the female at different temperature was translated in an age-specific cumulative probability distribution (Fig 2B). The curve of the three-parameter Weibull function described the distribution well ($F_{2, 367}$ = 5486.6; P < 0.0001; r^2 = 0.968) (Table 5). Parameter γ was 0.102 implying the female physiological age where they can start laying eggs and 50 % of eggs were laid when the female was aged 0.3515 ($\gamma + \eta$), physiologically.

Component models of Two-phase OM: PDRM and PCDM in POP and ODRM and OORM in OP. Pre-oviposition development of the female was well described with PDRM ($F_{3, 2}$ = 168.027; P = 0.00592; r^2 = 0.996) (Fig 3A, Table 5). Parameters, T_L and T_H, were estimated as 13.3°C and 35.2°C, respectively. The cumulative probability distribution of the females that completed pre-oviposition was slightly scattered, but the estimated curve of PCDM was well-fitted to the distribution ($F_{1, 389}$ = 20583.1; P < 0.0001; r^2 = 0.892) (Fig 3C, Table 5). Parameter γ was 0.4852 for the first occurrence of the females who completed POP, and 50% of the females completed POP when aged 0.9382 ($\gamma + \eta$). In OP, the ‘U’ shape of the female development rate was well-fitted to the curve of ODRM ($F_{3, 2}$ = 59.6469; P = 0.01653; r^2 = 0.989) (Fig 3B, Table 5). Cumulative oviposition probability distribution was well-described with OORM ($F_{1, 389}$ = 20583.1; P < 0.0001; r^2 = 0.981) (Fig 3D, Table 5). In total, 50% fecundity of eggs is laid at a physiological age of 0.2401 (parameter α).

Model simulation and comparison with observation

Both OMs, current OM and two-phase OM, predicted a daily egg production sequence laid by one female in a temperature range from 10°C to 40°C (Fig 5). Eggs were laid only between 17°C and 35°C in both models. However, the egg production of current OM lasted longer than that of two-phase OM. For example, in the case of two-phase OM, over 10 eggs were predicted at the 4th to 50th days after adult emergence, but current OM predicted this up to the 102nd day. Additionally, the output of two-phase OM (estimated 2) better matched the observed egg production in all observations than the output of current OM (estimated 1) as well as the pre-oviposition period in 16.7°C, 18.8°C, and 34.9°C (Fig 6, Table 6).

Discussion

Temperature ~~can affect the longevity of adults at an immature stage~~ [34, 46]. The aging process increases in terms of speed as temperature increases for adults. The longevity of the *B. dorsalis* adults becomes shorter (= ages faster) in increasing temperature conditions, and adults cannot live even a day over 52°C, which is the estimated maximum temperature (T_H) of the aging model for both sexes (Fig 1A and 1B, Table 1 and 4). On the other hand, the aging rate of both sexes slows as temperature decreases, and is stationary below 18.8°C, implying a minimum aging rate (≈ 0.01) per day for the adult, regardless of temperature. Given the estimated minimum aging rate (r_0) of the aging model, we expect that the average of the innate longevity ($1/r_0$) is 103 days for females and 107 days for males of *B. dorsalis*. The longevity of *B. dorsalis* observed by Yang et al [47] was longer than those observed in the present study. It is inferred that the difference between the two populations may be influenced by the food resources of the larvae and adults.

Reproduction is an important biological process that can accrue a number of individuals in a population. Mating is a prerequisite for reproduction, except for parthenogenesis, but harsh

environments like cold or hot temperatures hinder both ~~genders of adults~~ from mating with each other. Although we did not investigate their mating behavior and egg hatchability, we deduced a possible optimal temperature range for reproduction as no eggs were found at 13.5°C, where both *B. dorsalis* adults had survived for about 100 days, while eggs were observed in the other temperature treatments in this study (Table 1). Considering some of the eggs might be unfertilized, this observation infers that a suitable temperature for successful mating must be within a temperature range between 16.7°C and 34.9°C. Adults can emerge when the ground temperature is above 16°C and the optimum temperature is 22°C [15]. According to field occurrences, the optimal temperature for adult activity may range between 18~32°C [23, 48]. Additionally, there is a pre-copulation period and a specific period of mating time. For example, mating behavior starts 10 days after emergence and the mating rate reaches 50% at the 29th day and 80% at the 51st day, which is related to ovary development [49].

Bactrocera dorsalis females show a temperature-dependent development relationship for APOP and AOP (Fig 3A and 3B, Table 2). The optimal oviposition temperature range is likely between 18.8°C and 32.0°C because aging rates, reciprocals of the oviposition period, showed a proportional relationship to temperature only while it increased rapidly below or above the range. Fecundity did not show a linear relationship but is close to a symmetric campanula shape with a higher amount of eggs at 23.5°C and 28.8°C and the lowest at 16.7°C and 34.9°C at each side (Fig 2A, Table 5). Parameters, T_L and T_H, of the fecundity model were estimated as 16.3°C and 35.2°C, implying low and high temperature limitation for reproduction, respectively (Table 5). Daily egg production was dependent on temperature and most eggs were laid before the female aged to her half-life in that temperature (Fig 6). Cumulative egg production curves (Fig 2B, 3C and 3B) are a type of temperature-effect-removed distribution that depicts innate egg production based on the female's physiological age.

Many studies have modeled a linear relationship between insect development and

temperatures [50-51]. The LDT and TC of adults were estimated as 15.7°C and 433.4 DD for males and 16.0°C and 507.1 DD for females. TPOP is the generation time from the egg to when the fully grown adult lays its egg and whose LDT and TC were estimated as 10.6°C and 437.4 DD, which is similar to the LDT and TC of the Chinese population: 10.7°C and 501.7 DD [14]; and 12.2°C and 334.4 DD [20]. Although the degree-day model is simple for calculating their pivotal occurrence, it could not predict daily reproduction or even adult survival.

Curry and Feldman [39] suggested a basic structure of OM and Kim and Lee [35] developed the OM composed of a total fecundity model and two age-specific models, ORM and survival rate model, based on the physiological age derived from the female aging model (Fig 4A). This OM helps to predict the survival and progeny occurrence of a species with a short life span, such as a lepidoptera species [32-33, 40]. However, the logical structure of the current OM has no room to adopt a concept for the pre-oviposition period. For instance, *B. dorsalis* adult can live several months and have a considerable APOP before laying eggs. Therefore, APOP is long enough to affect female mortality and mating. Nonetheless, there is individual variance in the period. Furthermore, the current OM forced females complete APOP when aged 0.1020, as indicated by parameter γ of ORM. However, two-phase OM developed for *B. dorsalis* bypassed this problem and showed improved and more realistic simulation outputs according to the observations (Fig 5 and 6).

We showed a temperature effect on the longevity and fecundity of *B. dorsalis* adults reared with an artificial diet, although *B. dorsalis* females had a host preference for oviposition and their reproduction was affected by the host plants they had grown [29]. We improved the current OM using a two-phase OM and precisely estimated egg production under various temperatures. The actual egg populations from the field were not validated in this study but we can estimate the egg phenology in *B. dorsalis* and predict the lower and higher temperature thresholds of fecundity. In a previous study, we developed a temperature dependent

development model for *B. dorsalis* [25]. A population model structured by temperature dependent development and the new OM structure is helpful for predicting the egg production of *B. dorsalis* under field conditions and may be useful for understanding the population dynamics of this species.

Figure

Click here to access/download;Figure;figure1.TIF

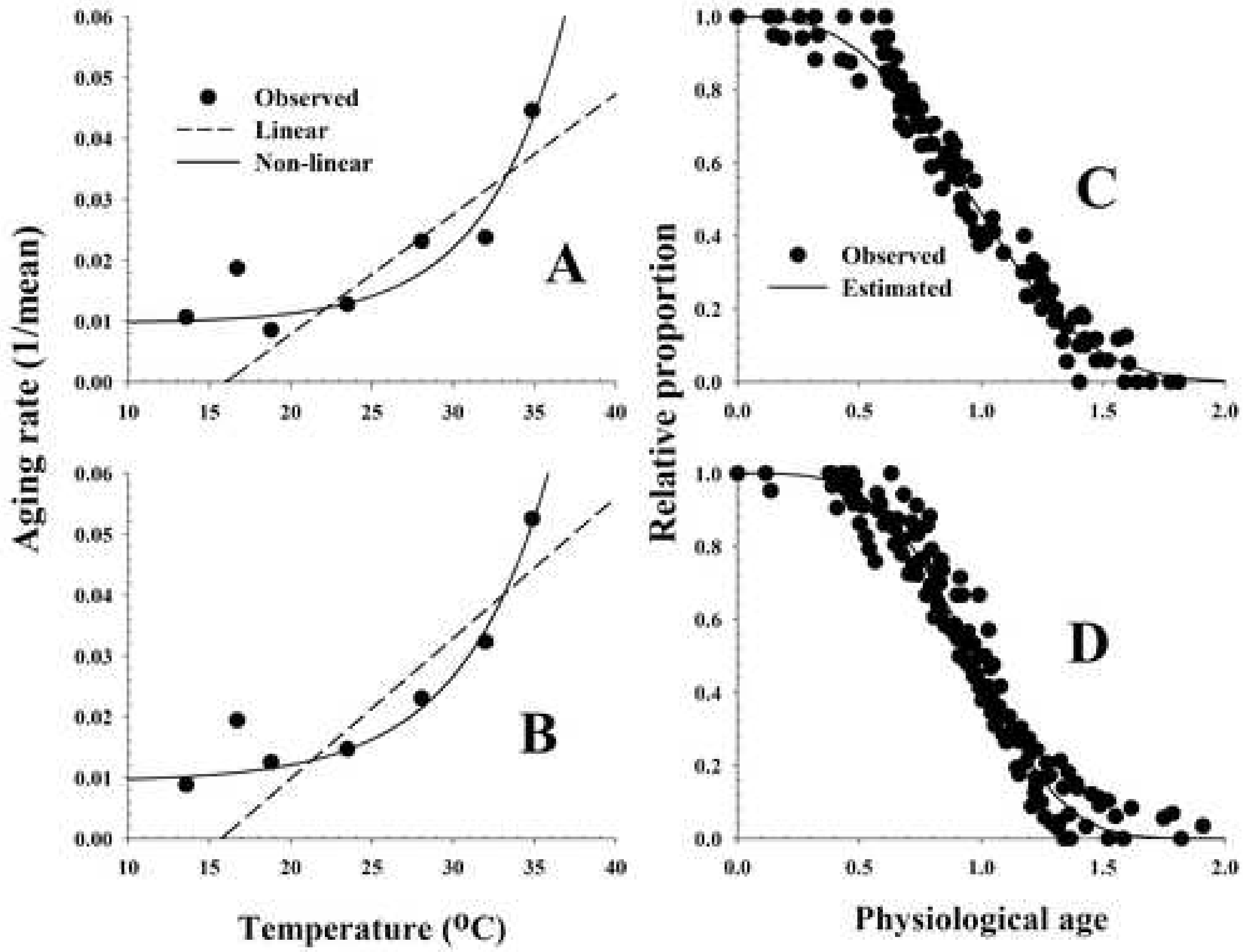

Click here to access/download;Figure;figure2.TIF

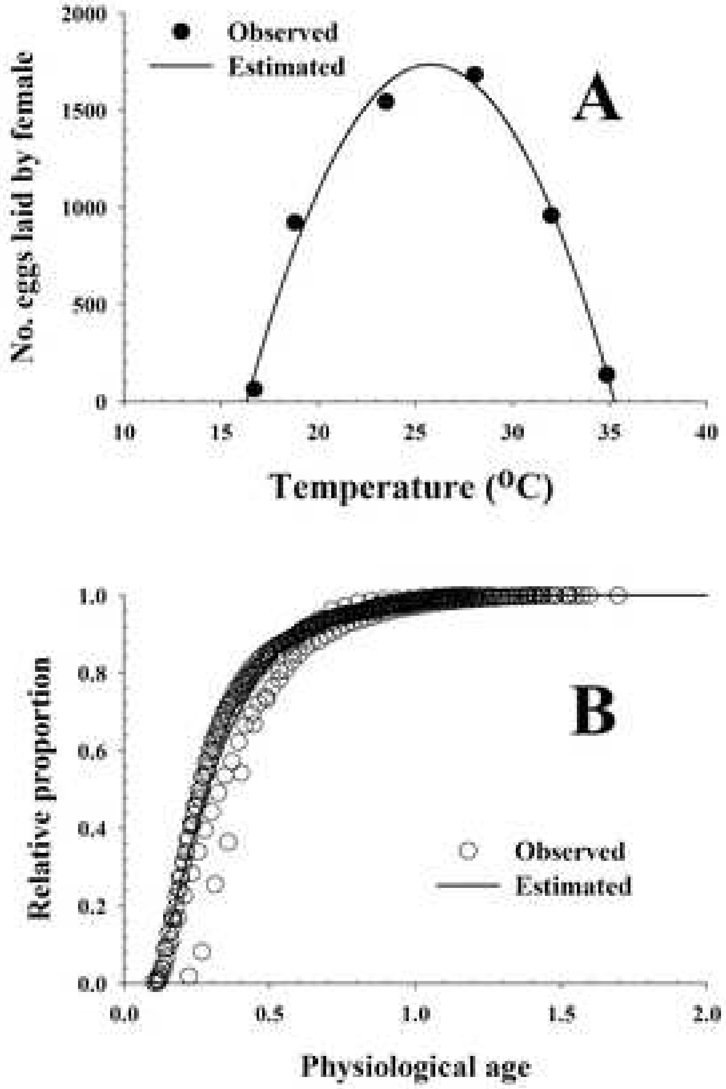

 Click here to access/download;Figure;figure3.TIF

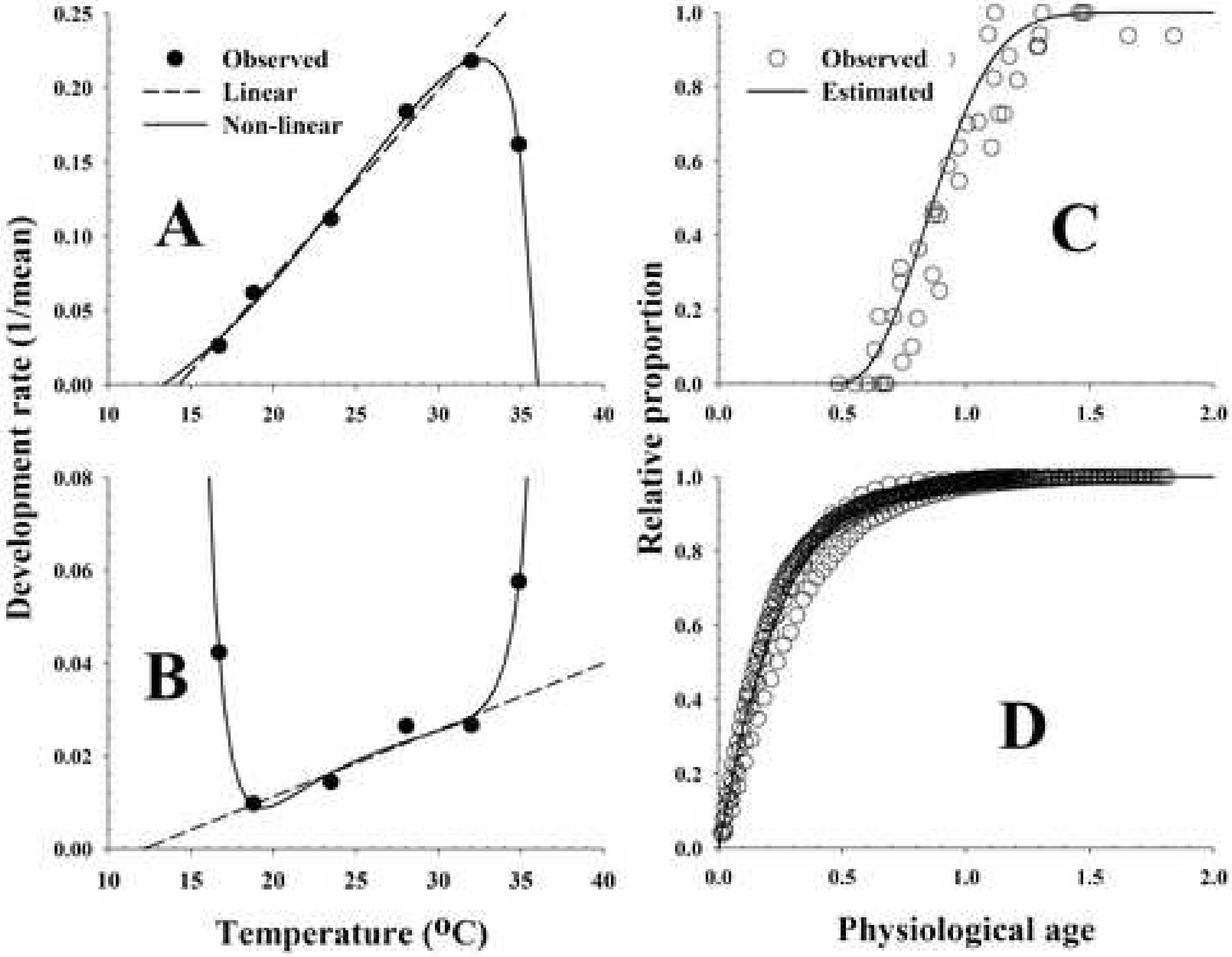

 Click here to access/download;Figure;figure 4.tif

A

B

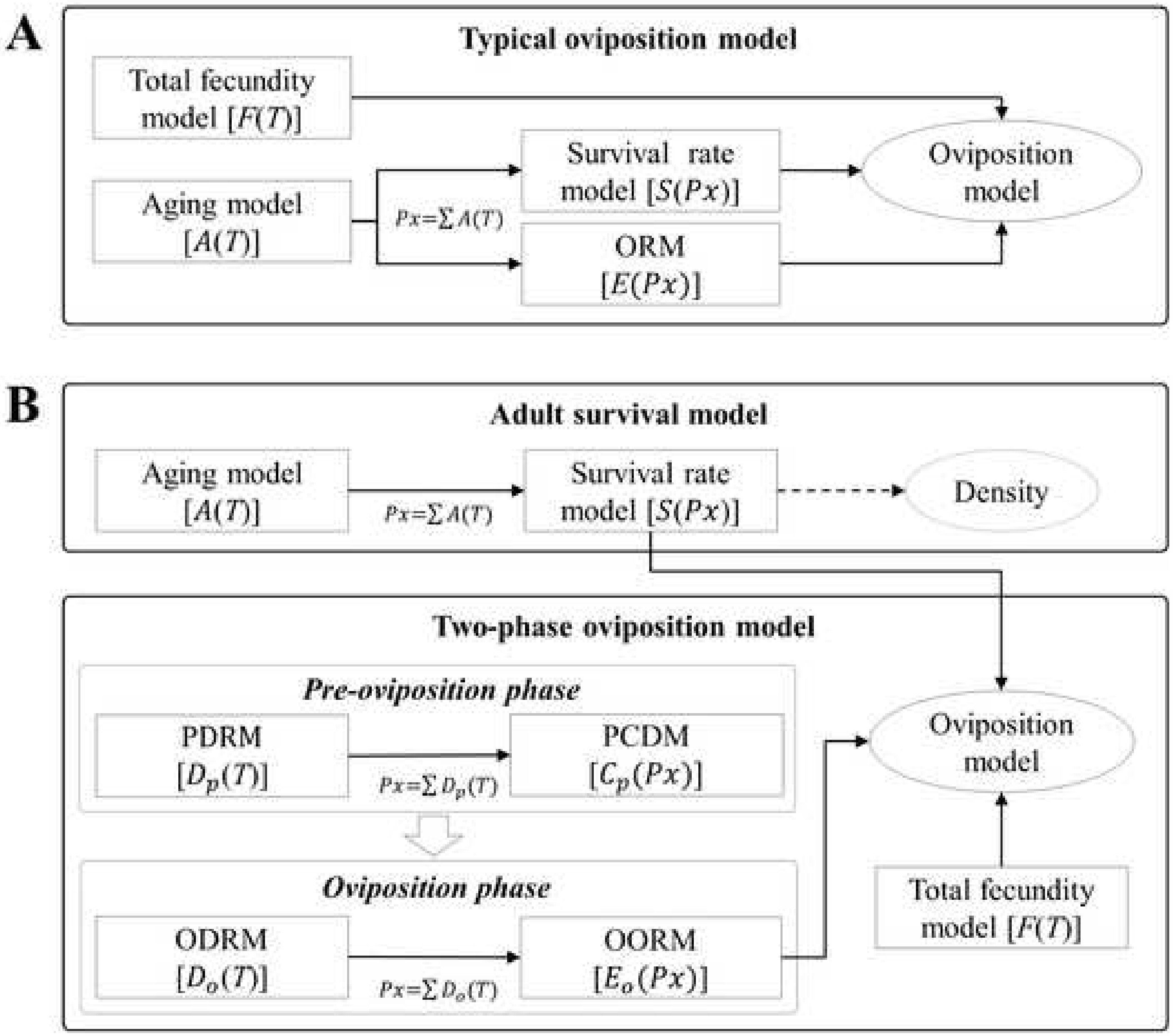

Click here to access/download;Figure;figure5.TIF

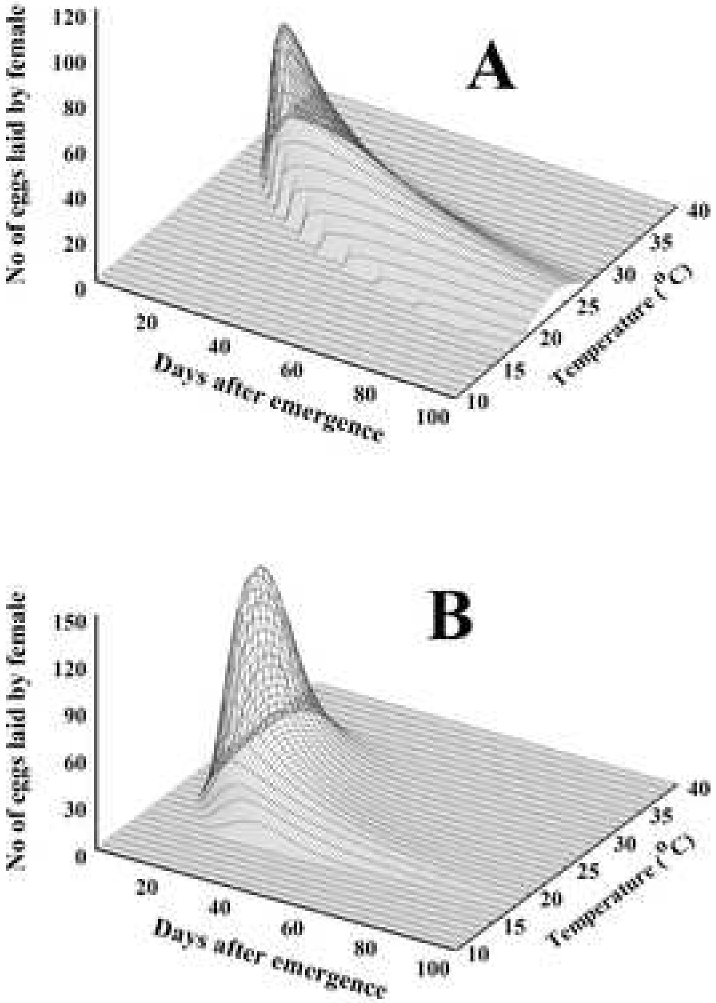

Figure

Click here to access/download;Figure;figure6.TIF

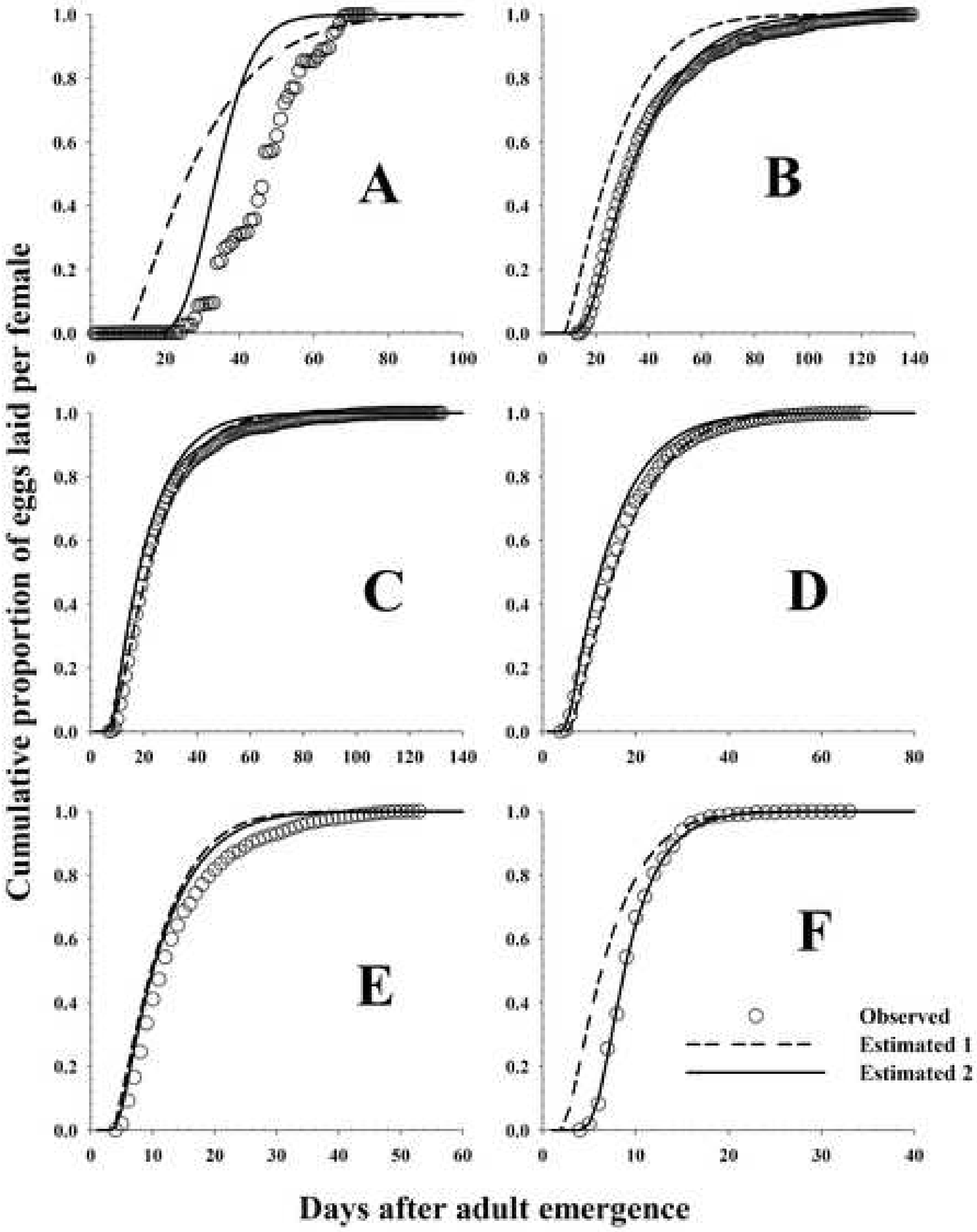

Supplement: Attachment — Submitted filename: PONE-D-20-03016_reviewer.pdf [file pone.0235910.s016.pdf]
