# Supplementary material for: Thermal effect on the fecundity and longevity of Bactrocera dorsalis adults and their improved oviposition model
Source: PLoS One. 2020 Jul 15;15(7):e0235910. doi: 10.1371/journal.pone.0235910 (PMC7363081; doi:10.1371/journal.pone.0235910)
Supplement: Attachment — Submitted filename: Response to Reviewers.docx [file pone.0235910.s017.docx]

**Response to Reviewers’ Comments**

All authors really appreciate two anonymous reviewers for valuable comments on our manuscript.

**Response to Editor’s Comments**

Thank you for ….these include:

**[RE] The authors appreciate your positive review.** We revised some parts of manuscript and tables.

1) **reduce/simplify the abbreviations and technical jargon**

**[RE] We revised it as suggested. Abbreviation “POP” and “OP” stand for pre-oviposition phase and oviposition phase.** Those are not general terms used in this kind of study but a term used to indicate separate phase in two-phase oviposition model of this study. **We removed those two abbreviations for reducing any confusion in the manuscript.** Other similar abbreviations, i.e. APOP, AOP, and TPOP, used in this manuscript. We introduce these abbreviations for compatibility with other studies because these abbreviations are often found other articles so that we leave them.

**Examples of recent article using APOP, AOP and TPOP**

Shi M-Z, Li J-Y, Ding B, Fu J-W, Zheng L-Z, Chi H. Indirect effect of elevated CO2 on population parameters and growth of *Agasicles hygrophila* (Coleoptera: Chrysomelidae), a biological agent of alligatorweed (Amaranthaceae). J Econ Entomol. 2019;112: 1120-1129.

Chen G-M, Chi H, Wang R-C, Wang Y-P, Xu Y-Y, Li X-D, Yin P, Zheng F-Q. 2018. Demography and uncertainty of population growth of *Conogethes punctiferalis* (Lepidoptera: Crambidae) reared on five host plants with discussion on some life history statistics. J Econ Entomol. 2018; 111: 2143-2152.

Liu J, Huang W, Chi H, Wang C, Hua H, Wu G. 2017. Effects of elevated CO2 on the fitness and potential population damage of *Helicoverpa armigera* based on two-sex life table. Scientific Reports. 2017;7: 1119.

Chen Q, Li N, Wang X, Ma L, Huang J-B, Haung G-U. Age-stage, two-sex life table of *Parapoynx crisonalis* (Lepidoptera: Pyralidae) at different temperatures. PLoS ONE. 2017:12; e0173380.

Oviposition models suggested by this study are composed of several unit models and have complicated structure. If reader has no background about this kind of works they may be confused the long and similar name. In order to minimize such confusion, we introduce simple abbreviations, i.e. ORM, PDRM, PCDM, ODRM, OORM, which would be better than their own name like Age-specific cumulative oviposition rate model. For readers, it is more convenient to use abbreviation in the manuscript.

2) **provide greater clarification on the approach (and the need for the approach) as well as the potential underlying physiology**

**[RE] We thank for pointing it out. Please refer to line 393-394.** This study is a part of series to build the phenology model of *Bactrocera dorsalis* to understand their occurrence in field and help protect their damage by predicting population dynamics. Although we studied the effects of temperature on the development of their immature stage, they spend their immature life inside fruit and soil and also immature period is relatively short (a couple of weeks). However, adult can survive for several months depending on temperature conditions. Therefore, atmospheric temperature can affect their life history like mating, oviposition, damage to fruit, spread and distribution. In this study, we examined longevity of male and female, and oviposition of female under different temperature conditions. Furthermore, we developed an improved oviposition model, two-phase oviposition model for the first time. Maintaining genome integrity is one of important factors in longevity and cell viability. Telomere length regulation is an important aspect of cell maintenance in eukaryotes (Hasty et al. 2003; Rodier e al. 2005). Various molecular mechanisms are likely to cause the specific telomere dynamics, including cell division, oxidative stress and telomerase activity (Jemiellty et al. 2007). Additional studies on the relationship between demographic and physiological approach for understanding and predicting the aging of insect are still needed.

Hasty P, Campisi J, Hoeijmakers J, van Steeg H, Vijg J. Aging and genome maintenance: lessons from the mouse? Science. 2003;299: 1355-1359.

Jemiellty S, Kimura M, Parker KM, Parker JD, Cao X, Aviv A, Keller L. Short telomeres in short-lived males: what are the molecular and evolutionary causes? Ageing Cell. 2007;6: 225-233.

Rodier F, Kim S-H, Niijar T, Yaswen P, Campisi J. Cancer and aging: the importance of telomeres in genome maintenance. The international Journal of Biochemistry & Cell Biology. 2005;37: 977-990.

**3) answer specific questions from Reviewer 2 re the experimental conditions used (see attached pdf)**

**[RE] We respect the comments. We give the answers from Reviewer 2’s questions.**

**Experimental Condition**

**Question 1: It is not clear whether flies were set up all at once, or was a 1 female: 2 males set up for each treatment over several days.**

**[RE] We have three growth chambers for experiment.** When we start the experiment at each temperature treatment we set up all replication within a day with one female and two males.

**Question 2: Table 2 shows the sample size to be 11-20 females per temperature. Eleven replicates seem on the low side**

**[RE] We thank for pointing it out.** The experiment started with 18 or 20 cages including one female and two males per cage. When we check the number of eggs and survivorship of male and female in each cage, adult can escape from the cage. We excluded all examined data related with the escaped adults. Therefore, in table 1, “n” means the number of female that we examined successfully during the experiment without any incident that lost female in a cage. In table2, “no. female oviposited” means the number of female oviposited from female of table 1.

**Question 3: Line 106 – Total pre-ovipositional period include data from another paper. What were the immature develop periods, this might be shown in the Table 2 as another column. Also, if this other data is added, were the conditions of measuring development similar?**

**[RE] We thank for pointing it out.** Total pre-ovipositional period (TPOP) was not examined from this study. TPOP deduced by summing APOP and development period of immature stage. We provide TPOP values for a reader who will need from this and our previous studies. The recorded temperature difference between this and previous study are less than 0.5°C except 20°C (0.7°C).

**4) address how variable temperature conditions have affected other species**

**[RE] We respect the comment. Please refer to line 405-406.** Insects are ectotherm and the effect of temperature on the development, longevity, fecundity and distribution of ectotherms has been well studied. Effects of temperature on those performances are different from species and life stages even though it is a same temperature. Specially, the current oviposition model has been developed for various insects and mites such as *Carposina sasakii* Matsumura (Kim and Lee 2003), *Tetranychus urticae* Koch (Kim and Lee 2003), *Otiorhynchus sulcatus* (F) (Son and Lewis 2005), *Scotinophara lurida* (Burmeister) (Kim and Lee 2008), *Riptortus pedestris* (Thunberg) (Kim et al. 2009, Ahn et al. 2019), *Neoseiulus californicus* (McGregor) (*Kim et al. 2013*), *Cnaphalocrocis medinalis* Guenée (Park et al. 2014), *Trissolcus basalis* Wholaston (Forouzan et al., 2015), *Ephestia kuehniella* (Zeller) (Pakyari et al. 2016), and *Rhopalosiphum padi* (Linnaeus) (Park et al. 2017). The temperature range of the highest total fecundity for each species was from 20°C to 25.2°C: 20°C for *C. sasakii*, 20.3°C for *R. padi,* 21°C for *O. sulcatus,* 24°C for *N. californicus,* 25°C for *R pedestris*, *T. basalis* and *E. kuehniella*, and 25.2°C for *C. medinalis*. The oviposition model of *C. sasakii* was incorporated into the population model of an orchard system for establishing management strategies (Kim and Lee 2010).

Kim D-S, Lee J-H. Oviposition model of *Carposina sasakii* (Lepidoptera: Carposiniae). Ecol Model. 2003;162: 145-153. <https://doi.org/10.1016/s0304-3800(02)00402-7>.

Kim D-S, Lee J-H. Oviposition model of overwintered adult *Tetranychus urticae* (Acari: Tetranychidae) and mite phenology on the ground cover in apple orchards. Experimental and Applied Acarology. 2003;31: 191-208.

Son Y, Lewis EE. Effects of temperature on the reproductive life history of the black vine weevil, *Otiorhynchus sulcatus*. Entomol Exp Appl. 2005;114: 15-24.

Kim H, Lee J-H. Phenology simulation model of *Scotinophara lurida* (Hemiptera: Pentatomidae). Environ Entomol. 2008;37: 660-669.

Kim H, Baek S, Kim S, Lee S-Y, Lee J-H. Temperature-dependent development and oviposition model of *Riptortus clavatus* (Thunberg) (Hemiptera: Alydidae). Appl Entomol Zool. 2009;44: 515-523.

Ahn JJ, Choi KS, Koh S. Effects of temperature on the development, fecundity and life table parameters of *Riptortus pedestris* (Hemiptera: Alydidae). Appl Entomol Zool. 2019;54: 63-74. <https://doi.org/10.1007/s13355-018-0593-5>.

Kim T, Ahn JJ, Lee J-H. Age- and temperature-dependent oviposition model of *Neoseiulus californicus* (McGregor) (Acari: Phytoseiidae) with *Tetranychus urticae* as prey. J Appl Entomol. 2013;137: 282-288.

Park H-H, Park C-G, Ahn JJ. Oviposition model of *Cnaphalocrocis medinalis* Guenee (Lepidoptera: Pyralidae). J Asia-Pac Entomol. 2014;17: 781-786.

Forouzan M, Shirazi J, Safaralizadeh MH, Safavi SA, Rezaei M. Oviposition model of *Trissolcus basalis* Wholaston (Hym.: Scelionidae) on sunn pest eggs. J Agr Sci Tech. 2015;17: 551-560.

Pakyari H, Amir-Maafi M, Moghadamfar Z. Oviposition model of *Ephestia kuehniella* (Lepidoptera: Pyralidae). J Econo Entomol. 2016;109: 2069-2073.

Park C-G, Choi B-R, Cho JR, Kim J-H, Ahn JJ. Thermal effects on the development, fecundity and life table parameters of *Rhopalosiphum padi* (Linnaeus) (Hemiptera: Aphididae) on barley. J Asia-Pac Entomol. 2017;20: 767-775.

Kim D-S, Lee J-H. A population model for the peach fruit moth, *Carposina sasakii* Matsumura (Lepidoptera: Carposinidae), in a Korean orchard system. Ecol Model. 2010;221: 268-280.

**Response to Reviewer 1’s Comments**

In this manuscript …improve the manuscript.

**[RE] The authors appreciate your positive review.** We revised some parts of manuscript.

There is an abrupt transition… a modified Kim and Lee model.

**[RE] We thank for pointing it out. Please refer to line 66-80.** We change the order of figures for explaining the modified model.

Aging as rate …..what is going on.

**[RE] We respect the comment. Please refer to line 393-394.** In 1980s, physiological age concept was introduced to solve the problem as you mentioned the flies lived the same days under different temperature conditions. Aging model produce a mathematical function how insect takes old under different temperature conditions. Physiological age, a sum of those temperature-dependent rate values that female has experienced after adult emergence, was applied to represent age of the female cohort according to mathematical logic developed by Curry and Feldman (1987). Aging model is developed by 1/ mean longevity and it produce one static value. Such distribution in survival and oviposition, even though females has same age, are obtained by transforming survival and oviposition according to the mean longevity. Therefore, different biological process caused by factors that are not related with temperature were incorporated in age-specific survival rate models and age-specific cumulative oviposition model. This kind of model process has been developed regarding such physiological variation that reviewer indicated. Exactly ageing model produce a mean age of the cohort at a day after adult emergence. The variation of age-specific survival and cumulative oviposition distribution describe the variation (Fig 2C, 2D, 3B, 4C and 4D).

Maintaining genome integrity is one of important factors in longevity and cell viability. Telomere length regulation is an important aspect of cell maintenance in eukaryotes (Hasty et al. 2003; Rodier e al. 2005). Various molecular mechanisms are likely to cause the specific telomere dynamics, including cell division, oxidative stress and telomerase activity (Jemiellty et al. 2007). Additional studies on the relationship between demographic and physiological approach for understanding and predicting the aging of insect are still needed.

Hasty P, Campisi J, Hoeijmakers J, van Steeg H, Vijg J. Aging and genome maintenance: lessons from the mouse? Science. 2003;299: 1355-1359.

Jemiellty S, Kimura M, Parker KM, Parker JD, Cao X, Aviv A, Keller L. Short telomeres in short-lived males: what are the molecular and evolutionary causes? Ageing Cell. 2007;6: 225-233.

Rodier F, Kim S-H, Niijar T, Yaswen P, Campisi J. Cancer and aging: the importance of telomeres in genome maintenance. Int J Biochem Cell B. 2005;37: 977-990.

Generally, there is mix …..in the model description.

**[RE] We thank for pointing it out. We revised it as suggested.** Abbreviation “POP” and “OP” stand for pre-oviposition phase and oviposition phase. Those are not general terms used in this kind of study but a term used to indicate separate phase in two-phase oviposition model of this study. We removed those two abbreviations for reducing any confusion in the manuscript. Other similar abbreviations, i.e. APOP, AOP, and TPOP, used in this manuscript. We introduce these abbreviations for compatibility with other studies because these abbreviations are often found other articles so that we leave them.

**Examples of recent article using APOP, AOP and TPOP**

Shi M-Z, Li J-Y, Ding B, Fu J-W, Zheng L-Z, Chi H. Indirect effect of elevated CO2 on population parameters and growth of *Agasicles hygrophila* (Coleoptera: Chrysomelidae), a biological agent of alligatorweed (Amaranthaceae). J Econ Entomol. 2019;112: 1120-1129.

Chen G-M, Chi H, Wang R-C, Wang Y-P, Xu Y-Y, Li X-D, Yin P, Zheng F-Q. 2018. Demography and uncertainty of population growth of *Conogethes punctiferalis* (Lepidoptera: Crambidae) reared on five host plants with discussion on some life history statistics. J Econ Entomol. 2018; 111: 2143-2152.

Liu J, Huang W, Chi H, Wang C, Hua H, Wu G. 2017. Effects of elevated CO2 on the fitness and potential population damage of *Helicoverpa armigera* based on two-sex life table. Scientific Reports. 2017;7: 1119.

Chen Q, Li N, Wang X, Ma L, Huang J-B, Haung G-U. Age-stage, two-sex life table of *Parapoynx crisonalis* (Lepidoptera: Pyralidae) at different temperatures. PLoS ONE. 2017:12; e0173380.

Oviposition models suggested by this study are composed of several unit models and have complicated structure. If reader has no background about this kind of works they may be confused the long and similar name. In order to minimize such confusion, we introduce simple abbreviations, i.e. ORM, PDRM, PCDM, ODRM, OORM, which would be better than their own name like Age-specific cumulative oviposition rate model. For readers, it is more convenient to use abbreviation in the manuscript.

The discussion cold benefit… fluctuating temperature versus constant temperatures.

**[RE] We respect the comment. Please refer to line 405-406.** The advantage of considering constant temperature is to measure direct effects of temperature tested on the development, longevity and fecundity. There have been debating on this subject because temperature fluctuated in nature and insects survive in there. Fortunately, insect needs a certain physiological event such as maturation and molting for developing not like a plant that required a change in temperature in a day. Many temperature dependent models including degree-days and non-linear models have been developed by using the results obtained under constant temperature conditions and also those models have been used practically for forecasting insect pest occurrence and establishing the management strategies. Therefore, we developed Degree-day models of APOP, AOP, and TPOP and modified the oviposition model using the data obtained under constant temperature conditions.

Wu et al. (2015) tested an additive model to predict the effects of fluctuating temperature on development and compared the development rate between the variable temperature and the static temperature treatments using published studies. Authors found the model was inadequate for making quantitative predictions but it was possible to explain some qualitative predictions such as a positive or negative effect. Authors mentioned “Development rate was faster under the variable temperature treatment than in the static temperature treatment as predicted by the additive model”. Lyons et al. (2013) conducted stable and fluctuating temperature effects on the development rate of two malaria vectors, *Anopheles arabiensis* and *Anopheles funestus* using nine constant and two fluctuating temperatures. They found development rate and survival of *An. funestus* was negatively influenced by fluctuating temperatures. On the other hand, development rate of *An. arabienssis* at fluctuating temperatures either did not differ from constant temperatures or was significantly faster. Kingsolver et al. (2015) pointed out that mean thermal performance can differ in fluctuating and constant thermal environments because thermal performance curves are non-linear. They showed *Manduca sexta* L. larvae reared in diurnally fluctuating temperatures had significantly higher optimal temperatures and maximal growth rates than larvae reared in constant temperatures.

Lyons CL, Coetzee M, Chown SL. Stable and fluctuating temperature effects on the development rate and survival of two malaria vectors, Anopheles arabiensis and Anopheles funestus. Parasites & Vectors. 2013;6: 104

Wu T-H, Shiao S-F, Okuyama T. Development of insects under fluctuating temperature: a review and case study. J Appl Entomol. 2015;139: 592-599.

Kingsolver JG, Higgins JK, Augustine KE. Fluctuating temperatures and ectotherm growth: distinguishing non-linear and time-dependent effects. Journal of Experimental Biology. 2015;218: 2218-2225.

**Response to Reviewer 2’s Comments**

This paper measures … period of the species.

**[RE] The authors appreciate your positive review.**

While the introduction is succinct ….is an improvement.

**[RE] We thank for pointing it out.** We revised it as suggested. **Please refer to line 66-80.**

Many of the points in paragraph 363-367 ….in the introduction

**[RE] We thank for pointing it out.** We revised it as suggested.

Many acronyms are used …..such as Adult POP, Adult OP, Total POP

**[RE] We respect the comment.** We revised it as suggested. Abbreviation “POP” and “OP” stand for pre-oviposition phase and oviposition phase. Those are not general terms used in this kind of study but a term used to indicate separate phase in two-phase oviposition model of this study. We removed those two abbreviation for reducing any confusion in the manuscript. Other similar abbreviations, i.e. APOP, AOP, and TPOP, used in this manuscript. We introduce these abbreviations for compatibility with other studies because these abbreviations are often found other articles so that we leave them.

**Examples of recent article using APOP, AOP and TPOP**

Shi M-Z, Li J-Y, Ding B, Fu J-W, Zheng L-Z, Chi H. Indirect effect of elevated CO2 on population parameters and growth of *Agasicles hygrophila* (Coleoptera: Chrysomelidae), a biological agent of alligatorweed (Amaranthaceae). J Econ Entomol. 2019;112: 1120-1129.

Chen G-M, Chi H, Wang R-C, Wang Y-P, Xu Y-Y, Li X-D, Yin P, Zheng F-Q. 2018. Demography and uncertainty of population growth of *Conogethes punctiferalis* (Lepidoptera: Crambidae) reared on five host plants with discussion on some life history statistics. J Econ Entomol. 2018; 111: 2143-2152.

Liu J, Huang W, Chi H, Wang C, Hua H, Wu G. 2017. Effects of elevated CO2 on the fitness and potential population damage of *Helicoverpa armigera* based on two-sex life table. Scientific Reports. 2017;7: 1119.

Chen Q, Li N, Wang X, Ma L, Huang J-B, Haung G-U. Age-stage, two-sex life table of *Parapoynx crisonalis* (Lepidoptera: Pyralidae) at different temperatures. PLoS ONE. 2017:12; e0173380.

Oviposition models suggested by this study are composed of several unit models and have complicated structure. If reader has no background about this kind of works they may be confused the long and similar name. In order to minimize such confusion, we introduce simple abbreviations, i.e. ORM, PDRM, PCDM, ODRM, OORM, which would be better than their own name like age-specific cumulative oviposition rate model. For readers, it is more convenient to use abbreviation in the manuscript.

Methods

It is not clear whether ……..each treatment over several days

**[RE] We thank for pointing it out.** We have three growth chambers for experiment. When we start the experiment at each temperature treatment we set up all replication within a day with one female and two males.

Table 2 shows the sample size to be ……on the low side.

**[RE] We thank for pointing it out.** The experiment started with 18 or 20 cages including one female and two males per cage. When we check the number of eggs and survivorship of male and female in each cage, adult can escape from the cage. We excluded the number of escaped adult from adding the number of female oviposited and survived in the table 1 and 2. Therefore, in table 1, “n” means the number of female that we examined during the experiment without losing female. In table2, “no. female oviposited” means the number of female oviposited from female of table 1.

Line 106 – Total pre-oviposition period …measuring development similar?

**[RE] We respect the comment.** Total pre-ovipositional period (TPOP) was not examined from this study. TPOP deduced by summing APOP and development period of immature stage. We provide TPOP values for a reader who will need from this and our previous studies. The recorded temperature difference between this and previous study are less than 0.5°C except 20°C (0.7°C).

Fixed temperatures are used in models,………as fixed temperatures

**[RE] We thank for pointing it out.** The advantage of considering constant temperature is to measure direct effects of temperature tested on the development, longevity and fecundity. Although we can measure the same independent variables under fluctuating temperature we know the effect of temperature range on the development, longevity and fecundity. There have been debating on this subject because temperature fluctuated in nature and insects survive in there. Fortunately, insect needs a certain physiological event such as maturation and molting for developing not like a plant that required a change in temperature in a day. Many temperature dependent models including degree-days and non-linear models have been developed by using the results obtained under constant temperature conditions and also those models have been used practically for forecasting insect pest occurrence and establishing the management strategies. Therefore, we developed Degree-day models of APOP, AOP, and TPOP and modified the oviposition model using the data obtained under constant temperature conditions.

Wu et al. (2015) tested an additive model to predict the effects of fluctuating temperature on development and compared the development rate between the variable temperature and the static temperature treatments using published studies. Authors found the model was inadequate for making quantitative predictions but it was possible to explain some qualitative predictions such as a positive or negative effect. Authors mentioned “Development rate was faster under the variable temperature treatment than in the static temperature treatment as predicted by the additive model”. Lyons et al. (2013) conducted stable and fluctuating temperature effects on the development rate of two malaria vectors, *Anopheles arabiensis* and *Anopheles funestus* using nine constant and two fluctuating temperatures. They found development rate and survival of *An. funestus* was negatively influenced by fluctuating temperatures. On the other hand, development rate of *An. arabienssis* at fluctuating temperatures either did not differ from constant temperatures or was significantly faster. Kingsolver et al. (2015) pointed out that mean thermal performance can differ in fluctuating and constant thermal environments because thermal performance curves are non-linear. They showed *Manduca sexta* L. larvae reared in diurnally fluctuating temperatures had significantly higher optimal temperatures and maximal growth rates than larvae reared in constant temperatures.

Lyons CL, Coetzee M, Chown SL. Stable and fluctuating temperature effects on the development rate and survival of two malaria vectors, *Anopheles arabiensis* and *Anopheles funestus*. Parasite Vector. 2013;6: 104

Wu T-H, Shiao S-F, Okuyama T. Development of insects under fluctuating temperature: a review and case study. J Appl Entomol. 2015;139: 592-599.

Kingsolver JG, Higgins JK, Augustine KE. Fluctuating temperatures and ectotherm growth: distinguishing non-linear and time-dependent effects. J Exp Biol. 2015;218: 2218-2225.

Minor

Line 32: “the current model” 🡪 “the current model developed by Kim and Lee”.

Line 34-36: We revised it as suggested. **Please refer to line 34-36**.

Line 46: “due to quarantine issue” 🡪 “due to market loss or reduction”.

Line 49: “annual occurrences” 🡪 “periodic occurrences”.

Line 54: “china” 🡪 “China”.

Line 57: “host plants” 🡪 We revised it as suggested. **Please refer to line 57-58**.

Line 65: We revised it as suggested. **Please refer to line 66-80**.

Line 99: “reproducing” 🡪 delete. **Please refer to line 113**.

Line 116: We revised it as suggested. **Please refer to line 131.**

Line 139-141: We revised it as suggested. **Please refer to line 154-156**.

Line 269: We revised it as suggested. **Reduce the number of significant digits**.

Line 318: We revised it as suggested. **Please refer to line 338-339**.

Line 330: We revised it as suggested. **Please refer to line 350-351**.

Line 332: “genders of adults” 🡪 “sexes”. **Please refer to line 352**.

Line 362: “pivotal occurrence” 🡪 “occurrence”. **Please refer to line 383**

Line 363-365: We revised it as suggested.
