# Supplementary material for: Thermal effect on the fecundity and longevity of Bactrocera dorsalis adults and their improved oviposition model
Source: PLoS One. 2020 Jul 15;15(7):e0235910. doi: 10.1371/journal.pone.0235910 (PMC7363081; doi:10.1371/journal.pone.0235910)
Supplement: Attachment — Submitted filename: Response to Reviewers.docx [file pone.0235910.s018.docx]

**Response to Reviewers’ Comments**

All authors really appreciate two anonymous reviewers for valuable comments on our manuscript.

**Response to Editor’s Comments**

Thank you for ….this letter:

**[RE] The authors appreciate your positive review.** We revised some parts of manuscript, tables and figures.

**Response to Reviewer 3’s Comments**

I think this is …section.

**[RE] The authors appreciate your positive review.** We revised some parts of manuscript, tables and figures.

Main comments:

Introduction …at different temperatures

**[RE] We thank for pointing it out. Please refer to line 81-84. We revised it as suggested.**

Materials & Methods

A higher detail …between studies

**[RE] We respect the comment. Please refer to line 92-94. W**e brought the insects (it may >10000 pupae) several times from TARI since 2016. We settle down the insect population in a new rearing system. As previous study [26], we used yellow cylindrical cups to collect the eggs deposited by the adult female. After hatching, larvae were reared on an artificial diet. Sawdust was provided as pupation substrate. The pupae were collected by sieving the sawdust. The pupae moved to a 30 x 30 cm container where the adults emerged. From egg to adult emergence, it took about two weeks under laboratory system (25±2°C). According to our unpublished observation and other references, adults can survive several months in room temperature (26±1°C). Female adults lay more than 1000 eggs during their whole life in the same temperature conditions. There may be 10 generations per year while the adults from the first or second generation may be alive in laboratory condition. It is very hard to explain the exact density, number of generations and how their colony are mixed in the laboratory condition.

Results

Some figures … comments below

**[RE] We thank for pointing it out. We revised it as suggested.** **Please refer to figures.**

Discussion

In the discussion… comment below.

**[RE] We respect the comment. Please refer to line 411-412.**

**One additional point… in insects.**

**[RE] We thank for pointing it out.** Son and Lewis (2005) conducted a laboratory experiment to quantify the stage-specific effects of temperature on development time and survival of *Otiorhynchus sulcatus*. They quantified the stage-specific development of *O. sulcatus* immature stages at constant temperatures and demonstrated that the range of temperatures at which development and survival occurred was different among developmental stages. We only investigated the longevity and fecundity of *B. dorsalis* adults in this study. We did the experiments in previous study [26]. It is not possible to explain the relationship between oviposition model and the variation of survival rate across juvenile developmental stages.

**Specific comments**

Line 58: Replace feed by “feeds”. 🡪 We revised it as suggested. **Please refer to line 58**.

Line 60: Re-phrase “… inferred from studies on the relationship..” 🡪 We revised it as suggested. **Please refer to line 60.**

Line 64: Re-phrase “… oviposition modeling including modeling of the immature...”🡪 🡪 We revised it. **Please refer to line 63-64.**

Lines 67-71: If you decide…the species 🡪 We revised it as suggested. **Please refer to line 67-69.**

Line 71: Re-phrase “…OM comprises three…” 🡪 **Please refer to line 70.**

Line 72: Change to “These two age-specific models….” 🡪 **Please refer to line 71-72.**

Lines 72-76: These sentences……their meaning. 🡪 **Please refer to line 71-74.** Original ovipostion model developed by Kim and Lee (2003) is complicated because they use “physiological age” as calculation term. We describe the structure of the original oviposition model and how temperature used as an input value and how physiological age is calculated as an independent variable.

Line 78: “have” instead of “has” 🡪 **Please refer to line 77.**

Line 82 - why is this new model … previous model(s) 🡪 We revised it. **Please refer to line 81-83.**

Line 91: Some more …. laboratory culture? 🡪 **Please refer to line 92-94. W**e brought the insects (it may >10000 pupae) several times from TARI since 2016. We settle down the insect population in a new rearing system. As previous study [26], we used yellow cylindrical cups to collect the eggs deposited by the adult female. After hatching, larvae were reared on an artificial diet. Sawdust was provided as pupation substrate. The pupae were collected by sieving the sawdust. The pupae moved to a 30 x 30 cm container where the adults emerged. From egg to adult emergence, it took about two weeks under laboratory system (25±2°C). According to our unpublished observation and other references, adults can survive several months in room temperature (26±1°C). Female adults lay more than 1000 eggs during their whole life in the same temperature conditions. There may be 10 generations per year while the adults from the first or second generation may be alive in laboratory condition. It is very hard to explain the exact density, number of generations and how their colony are mixed in the laboratory condition.

Line 112: please provide … sample sizes 🡪 **Please refer to line 117-119.**

Lines 133-134: Provide a brief definition of LDT and TC here. 🡪 **Please refer to line 140-143.**

Line 193: briefly explain… aging model (lines 146). 🡪 **Please refer to line 203.**

Line 208: same as commented above for line 193 🡪 **Please refer to line 218-219.**

Line 274: Change to “…period of the females varied across temperatures 🡪 **Please refer to line 284-285.**

Line 365: This is the first time… the discussion. 🡪 **Please refer to line 374-375.**

Line 380: same as commented above for line 365 🡪 **Please refer to line 391.**

Lines 397-399: Some aspects … much worth discussing 🡪 **Please refer to line 411-412.**

Table 2: correct reference is 25 or 26 🡪 **Please refer to line 717 and 726 .**

Figure 2: complete legend of X axis – “Aging rate (1/mean of… 🡪 **Please refer to figure 2. (1/mean of adult longevity).**

Figure 4: complete legend of X axis– “Development rate (1/mean of…)” 🡪 **Please refer to figure 4. (1/mean of female development period).**

Figure 5: Put each model name in the title (above the figure) 🡪 **Please refer to figure 5.**

Figure 6: replace “estimated 1”, “estimated 2” by the models' names .. possible refer the temperature analyzed in each graph 🡪 **Please refer to figure 6.**
